# Genomic Analysis of Oral Lichen Planus and Related Oral Microbiome Pathogens

**DOI:** 10.3390/pathogens9110952

**Published:** 2020-11-16

**Authors:** Evelyn F. Zhong, Andrea Chang, Andres Stucky, Xuelian Chen, Tarun Mundluru, Mohammad Khalifeh, Parish P. Sedghizadeh

**Affiliations:** Diagnostic Sciences, Herman Ostrow School of Dentistry, University of Southern California, Los Angeles, CA 90089, USA; chan521@usc.edu (A.C.); astucky@usc.edu (A.S.); xuelianc@usc.edu (X.C.); tmundlur@usc.edu (T.M.); khalifeh@usc.edu (M.K.)

**Keywords:** T-helper cell, next generation sequence, microbiome, HNF4A, *Prevotella denticola*, lichen planus pathogenesis

## Abstract

Oral lichen planus (OLP) is a common chronic inflammatory disease affecting the oral mucosa. The pathogenesis of OLP is incompletely understood but is thought to be related to the immune system. As the oral cavity is a major reservoir and transmission gateway for bacteria, viruses, and fungi, the microbial composition of the oral cavity could play a role in the pathogenesis of OLP. However, limited by analytic technology and knowledge of the microbial community in the oral cavity, it is not yet clear which pathogens are associated with OLP. Next generation sequencing (NGS) is a powerful tool to identify pathogens for many infectious diseases. In this study, we compared the host cell gene expression profiles and the microbial profiles between OLP patients and matched healthy individuals. We identified the activation of the hepatocyte nuclear factor alpha (HNF4A) network in OLP patients and potential pathogens, including *Corynebacterium matruchotii*, *Fusobacterium periodonticum*, *Streptococcus intermedius*, *Streptococcus oralis*, and *Prevotella denticola*. *Prevotella denticola* is capable of activating the HNF4A gene network. Our findings shed light on the previously elusive association of OLP with various diseases like hepatitis, and indicate that OLP is a T-helper type 17 (Th17) mediated mucosal inflammatory process. The identified molecular pathways and microbes could be used to inform future investigations into OLP pathogenesis and to develop novel therapeutics for OLP treatment.

## 1. Introduction

Lichen planus is a common chronic mucocutaneous inflammatory disease affecting the skin, nails, eyes, urogenital tract, and oral mucosa [1]. Oral lichen planus (OLP) occurs specifically in the oral cavity. As a variant of lichen planus, OLP is believed to affect the oral mucosa by T-cell-mediated chronic inflammation, and some investigators suggest that Th2-mediated inflammation can also contribute to the pathogenesis of OLP [2]. 

Five clinical subtypes of OLP are usually seen: reticular, plaque-like, atrophic, erosive-ulcerative, and bullous. Symptomatic lesions can appear and regress at intervals. Cutaneous involvement in addition to oral mucosal lesions is seen clinically in a subset of patients [3]. The most commonly affected oral location in any of the types of OLP is the buccal mucosa, usually with symmetrical involvement [4]. This is followed by the gingiva, tongue, lip, and palate. OLP has a greater prevalence in females compared to males, with a more chronic course and higher potential for significant morbidity [5]. OLP also has a prevalence of approximately 0.5–2% with an age onset between 30 and 60 years old [3].

A major clinical challenge of OLP for clinicians is providing patients with successful management or treatment without negative side effects. Currently, one of the most common treatments for OLP is topical corticosteroids [6]. Topical therapy is commonly favored over systemic therapy as the former is easier and more cost-effective when compared to the latter. However, some patients do not respond to topical therapy. Systemic therapy often requires additional treatment such as concomitant topical therapy [7]. Moreover, the use of topical treatment without proper monitoring and evaluation can lead to oral candidiasis with associated burning mouth and hypogeusia, hypersensitivity reactions to the drug, inhibition of the hypothalamic-pituitary-adrenal axis and secondary adrenal insufficiency, or blood glucose dysregulation. Reports of patients with reactions to topical corticosteroids have also been detailed and highlight the clinical course and challenges in managing patients with OLP [8].

Therefore, investigating the related gene networks of host cells and the microbiome associated with OLP could have significant clinical impact in our understanding and ultimately treatment of OLP. In this study, we performed next generation sequence (NGS) to compare the host gene expression and microbial profiles between OLP patients and healthy controls. We identified specific host gene expression profiles of OLP as well as unique microbial profiles in OLP patients. These findings provide insights into potential OLP pathogenesis and could help inform future targeted therapies. 

## 2. Materials and Methods

### 2.1. Patient Population

Institutional review board approval was obtained for this study. Patient samples were obtained at the Herman Ostrow School of Dentistry, Oral Medicine Clinic, in Los Angeles, California. Inclusion criteria for OLP patients included males or females with biopsy and histologically confirmed lichen planus diagnosis by a board-certified oral pathologist, and the ability to provide informed consent. Exclusion criteria for OLP patients included xerostomia or significant polypharmacy where saliva collection could be difficult or prohibitive, active systemic infection or oral infection such as periodontitis or abscess, detectable subgingival or supragingival plaque, active antibiotic therapy, and patients with any history or skin involvement by lichen planus. Ascertainment criteria for controls included healthy male or female patients without a diagnosis of cutaneous or oral lichen planus in any form, one or no comorbidities, no xerostomia or polypharmacy, no active systemic or oral/periodontal infection, and no detectable subgingival or supragingival plaque. Periodontal status was assessed in all cases clinically by visual inspection of the gingiva and teeth for signs of plaque or calculus and plaque-related gingivitis (e.g., swelling and attachment loss), and by periodontal probing around several teeth in each quadrant. Disclosing solutions were not used for plaque identification, and all patients were patients of record at our dental school who had a dental hygiene appointment and cleaning or maintenance therapy within 3 months of sample collection, and who were referred to our oral medicine specialty clinic by our dental or periodontal clinics precisely because they were deemed to have good oral hygiene, regular plaque control, no active periodontal disease, but continued inflammation of their gingiva in a desquamative pattern. None of the patients were deemed to have active plaque-related periodontal disease/infection, which was required for study inclusion as indicated above, hence raising suspicion for an autoimmune condition warranting specialty evaluation for diagnosis and management. 

### 2.2. Sample Collection

This study was retrospective in nature, using samples already being collected for clinical purposes as per our IRB protocol. As such it represents a convenience sampling which has inherent bias and limitations as compared to prospective controlled data collection. Saliva and buccal mucosa wash samples of the patients diagnosed with OLP (n = 10) and healthy controls (n = 5) were collected in OMNIgene saliva collection kits (Genotek, Ottawa, Ontario, Canada). Saliva and buccal wash cells were collected from each patient under standardized conditions. Salivary flow rates vary significantly among individuals and in the same individual under different conditions or during different times of the day, thus patients were instructed not to eat, clean, or rinse their mouth 1 h before saliva collection, because these activities can affect the microbial environment. Saliva for all study subjects was collected at the same time of day by a draining method with the patient in an upright head position. Whole unstimulated saliva was collected for 5 min in a Proflow sialometer (Amityville, New York, NY, USA) or until 0.5 mL of saliva was collected (0.1 mL/min average). These samples were subsequently used for molecular studies. 

### 2.3. Next Generation Sequencing

DNA Libraries were constructed using Nextera DNA Flex Library Prep Kit (Illumina, San Diego, CA, USA). RNA-seq library was constructed using the NEBNext Ultra II RNA library prep kit for Illumina and the Ribo-Zero rRNA Removal Kit (NEB, Ipswich, MA, USA). RNAseq and DNAseq libraries were sequenced on the Illumina HiSeq 4000 platform (Illumina, San Diego, CA, USA). Results were analyzed using Partek Flow (Partek^®^ Genomics Suite^®^ software, version 7.0 Copyright ©; 2020 Partek Inc., St. Louis, MO, USA). To investigate the relationship between OLP and oral microbial status, we conducted RNA sequencing of patient saliva with buccal epithelial cells and aligned reads to human, bacterial and viral genomes. We performed an observational (clinical paired with genomic patient information) study to identify possible associations. 

### 2.4. Sequencing Data Analysis

The raw reads were filtered for minimum sequencing quality, duplicated reads and adaptor contamination. Bases with Phred scores <20 were trimmed from both ends of the raw sequencing reads and trimmed reads shorter than 25nt were excluded from downstream analyses. Both pre- and post-alignment quality assessment and quality control were carried out with default settings as part of the Partek Flow workflow. The retained high-quality reads were used in the genome assembly. The sequencing data were analyzed with Partek Flow (Partek^®^ Genomics Suite^®^ software, version 7.0 Copyright ©; 2020 Partek Inc., St. Louis, MO, USA.). Trimmed reads were mapped onto human genome hg38 using Tophat 2.0.8 [9] as implemented in Partek Flow with default settings, using Gencode 31 annotation as guidance (gencodegenes.org). Samples were normalized using transcripts per million (TPM). 

### 2.5. Differential Gene Expression Analysis

Pooled gene expression values from healthy or OLP patients’ transcriptomes were used for differential gene expression analysis. Partek’s Gene Specific Analysis method (Partek^®^ Genomics Suite^®^ software, version 7.0 Copyright ©; 2020 Partek Inc., St. Louis, MO, USA) was used to generate a list of significantly differentially expressed genes between OLP patient samples and controls (genes with <10 reads in any sample were excluded). Significance was determined with false discovery rate (FDR) adjusted *p*-value (q-value < 0.05), and >2-fold change difference in gene expression. Ingenuity Pathway Analysis (IPA) software (Qiagen Bioinformatics, Redwood City, CA, USA) was used to analyze gene-specific pathway [10]. Genes enriched in the most significant pathway (lowest *p*-values) were selected for evaluation of clinical data meta-analysis.

### 2.6. Microbial Profiling

Kraken is a taxonomic profiling tool [11] and was used to map non-human reads (reads do not map to human genome hg38) to microbial and viral genomes in GenBank, a NIH genetic sequence database [12]. Approximately 22 million non-human reads (paired-end, 150 bp) of each sample were used to map the GenBank microbiome database (>4000 bacterial genomes, >10,000 viral genomes.), and approximately 96.4% of input reads aligned with known microbiome genomes. Abundance of known microbiome species were calculated as normalized counts after normalization by genome size as described [11]. Shannon, Alpha and Beta diversities were estimated using the R package vegan (https://github.com/vegandevs/vegan) and there is no significant difference of microbiome diversity among all samples.

### 2.7. PCR Confirmation

An amount of 500 ng of total DNA was used to amplify and quantify the microbial genes present in the patients’ samples using a CFX Connect system (BIO-RAD), and ß-actin was used to quantify the amount of human cells in the DNA samples. Confirmed microbes identified with RT-PCR were normalized to ß-actin (human cell numbers). Primers were listed:

Corynebacterium matruchotii
Forward: TGGTGACGGTACCTTTGTTA
Reversed: CACCCTCACAGGTTAGCAGCGCTT
Fusobacterium periodonticum
Forward: CGCAGAAGGTGAAAGTCCTGTAT
Reversed: TGGTCCTCACTGATTCACACAGA
Streptococcus intermedius
Forward: AAGTAGAACGCACAGGATG
Reversed: CAGTAAATGTTCTTATGCGGTATTAG
Streptococcus oralis
Forward: ACCAGCAGATACGAAAGAAGCAT
Reversed: AGGTTCGGGCAAGCGATCTTTCT
Prevotella denticola (an amplicons size of 316 bp) (13).
Forward Primers: TAATACCGAATGTGCTCATTTACAT
Reversed primer: TCAAAGAAGCATTCCCTCTTCTTCTTA

## 3. Results

### 3.1. Clinical and Pathologic Features

**Figure 1:**
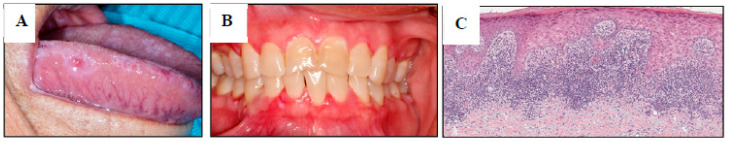
Clinical and pathologic features of oral lichen planus (OLP). (**A**) image shows OLP involving the tongue with characteristic white striae (reticular variant) and red atrophic lesions (erosive variant). (**B**) image shows OLP with inflamed and desquamative gingivitis. (**C**) Histopathology of lichen planus demonstrates the characteristic band-like inflammatory cell infiltrate subjacent to the epithelium with “saw-tooth” rete ridge morphology and hydropic degeneration of basal keratinocytes.

We studied 10 patients with OLP and five control patients without OLP; our OLP patient population had signs of both reticular and erosive patterns, and the most common sites of involvement were the buccal mucosa, gingiva, and tongue (Table 1). A common clinical sign of OLP is oral lesions with radiating whitish gray lines or thread-like papules, which can be lacy or reticular, annular, patches, or strings. The term Wickham’s striae is often used to describe the morphology of the white lace-like lesions, and desquamative gingivitis is also another common pattern for OLP (Figure 1A,B). When Wickham’s striae predominate clinically, the term reticular OLP is used. When atrophic or ulcerative lesions predominate clinically, the term erosive OLP is used. Significant clinical characteristics of OLP are lesions that often alternate between periods of exacerbation and quiescence, as was seen in our patient population [13]. Patients may notice an irritation to the oral cavity and “burning” sensations at a later period. The “burning” sensations can be further aggravated with the intake of spicy or hot foods and can interfere with patient eating habits and quality of life [14]. None of our OLP patients had skin involvement or other tissue involvement outside the oral cavity.

It is usually recommended to confirm the diagnosis of OLP with an oral biopsy and histopathological examination as lesions may mimic dysplasia or malignancy in addition to other conditions [15]. Thus, we confirmed OLP diagnosis in all studied patients via histopathologic evaluation. Histopathologic criteria for diagnosis include the presence of a well-defined, band-like zone of inflammatory cell or lymphocytic infiltration within the superficial part of the connective tissue or lamina propria (Figure 1C). Histologic evidence of hydropic degeneration in the basal cell layer with absence of epithelial dysplasia is also common [16]. The finding of “saw-tooth” shaped rete ridges and colloid bodies are other useful features which help support the diagnosis of OLP.

### 3.2. Molecular Analysis of OLP Related Gene Network

The pathology of OLP is strongly related to immune dysregulation, like any other autoimmune disease. The most popular theory is activated cytotoxic CD8+ cells target basal keratinocytes, where CD4+ helper T cells secrete TH1 cytokines [17]. Along with T cells, growth factors, inflammatory and proapoptotic mediators up and down regulate the inflammation [18]. However, as of now, there is no definitive etiology for OLP, but some evidence suggests viral or bacterial infections, local trauma or irritation, systemic disorders, and even excessive alcohol and tobacco consumption are probable factors [19].

**Figure 2:**
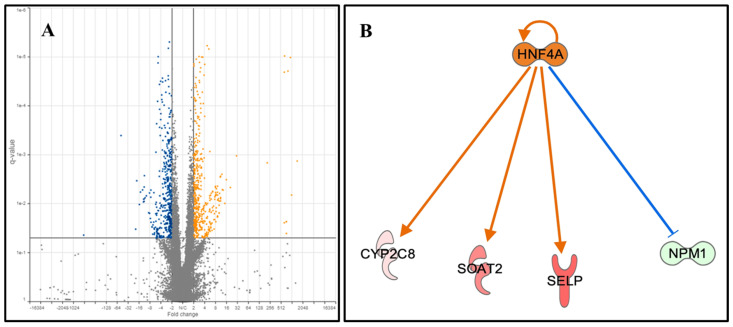
Differential expressed genes between OLP patients and healthy individuals. (**A**). Volcano plot illustrating differentially expressed genes identified between OLP patients and healthy controls. Y axis indicates false discovery rate (FDR)-adjusted p-values, while the x axis indicated the fold change between OLP and Controls. Overall, we identified 959 differentially expressed genes, of which 503 were upregulated and 456 were downregulated between OLP patients and controls. Further analysis of the differentially expressed genes identified hepatocyte nuclear factor 4 alpha (HNF4A) as a significant upstream regulator of the identified differentially expressed genes. (**B**). The hepatocyte nuclear factor alpha (HNF4A) gene network is activated in OLP patients. Differential gene expression analysis indicated that the HNF4A gene network is activated in OLP patients. Comparing to healthy individuals, HNF4A and its downstream targets, CYP2C8, SOAT2 and SELP are upregulated in OLP samples, while the inhibitor of HNF4A, NPM1 is downregulated in OLP compared to healthy individuals. Red: upregulation in OLP, Green: Downregulated in OLP.

Most of the current evidence supports the notion that OLP is related to immune dysregulation. However, the immune system is not only affected by drugs, systematic metabolic diseases, physical and mental stress, but also by the oral microbiome or pathogens. There are many factors which affect the immune system of a person. The oral cavity is the beginning of the gastrointestinal system, and the gut–body connection and microbiome play important roles in maintaining health versus disease oral cavity. Therefore, OLP is likely a result of multiple factors which stress or dysregulate the immune system. By comparing the gene expression profiles of cells in oral cavity, we identified 959 genes that showed significantly different expression profiles when lichen planus patients were compared to healthy controls (Table S1). Among the differentially expressed genes, we identified 503 up-regulated and 456 down-regulated genes (q < 0.05, fold change > 2) between OLP patients and controls (Figure 2A). Drawing from the gene expression profiles of study subjects (the set of 959 with expression profiles q < 0.05, fold change >2), we used Ingenuity Pathway Analysis (IPA^®^), to identify the most likely molecular signaling mechanisms that could account for some of the symptomatic manifestations of OLP. Among the OLP patients, from the enriched signaling pathways we identified several genes coding for proteins involved in nicotine degradation (*p* = 0.003), cysteine biosynthesis and homocysteine degradation (*p* = 0.015), as well pathways associated with increased activation of hepatocyte nuclear factor alpha (HNF4A) (*p* = 0.015) and the modulation of its various downstream signaling molecules (Figure 2B). HNF4A is highly expressed in lymphatic tissue and in the salivary glands, which is consistent with the observation of an activated HNF4A gene network. Among this gene network, the largest differences were observed in the 9-fold increased sterol O-acyltransferase 2 gene (SOAT2) (*p* = 0.006), a protein involved in cholesterol metabolism that has been implicated in various types of cancer including epithelial and liver cancer as well as hepatocellular carcinoma, melanoma, and acute myeloid leukemia. In addition to SOAT2, CYP2C8 was upregulated (2-fold) (*p* = 0.03) and is an unspecific monoxygenase. Selectin P (SELP) was also upregulated (2-fold, *p* = 0.004) and is a cell adhesion protein upregulated in response to bacterial infections, delayed hypersensitivity reaction, T-cell lymphoma, and systemic lupus erythematosus. The inhibitor of HNF4A, NPM1 is down-regulated in OLP compared to healthy individuals. These results suggest that HNF4A is activated in OLP patients and contributes to OLP pathogenesis.

### 3.3. Microbes Associated with OLP

**Figure 3:**
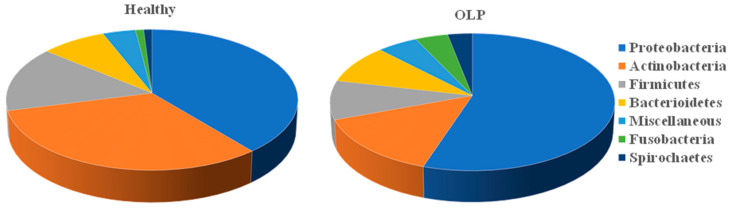
Relative abundance of identified bacterial and archaea species sequences in the saliva of patients with OLP and healthy controls. Among all samples, 1390 different microbiome species were identified. Of the identified microorganisms, 25 bacterial species were observed to be differentially expressed between the OLP patients and healthy controls. The top 7 bacterial phyla are compared and plotted. OLP samples have more Proteobacteria (54.7% vs. 39.0%), Fusobacteria (4.3% vs. 0.6%) and Spirochaetes (3.5% vs. 1.2%), but with less Actinobacteria (14.3% vs. 32.0%) and Firmicutes (9.7% vs. 15.0%).

**Figure 4:**
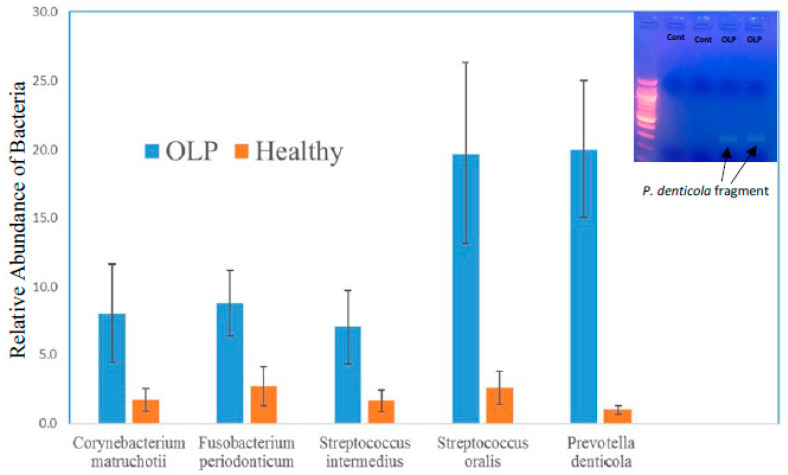
PCR confirmation of *Corynebacterium matruchotii*, *Fusobacterium periodonticum*, *Streptococcus intermedius*, *Streptococcus oralis* and *Prevotella denticola* are dominant bacteria in OLP patients (OLP: n = 10, Cont: n = 5). As a representative case, PCR primers designed to amplify a 316bp fragment of *Prevotella denticola* demonstrate the present of this bacteria in two OLP patients but not in matched healthy controls (Inset). Error bars represent s.e.m.

**Figure 5:**
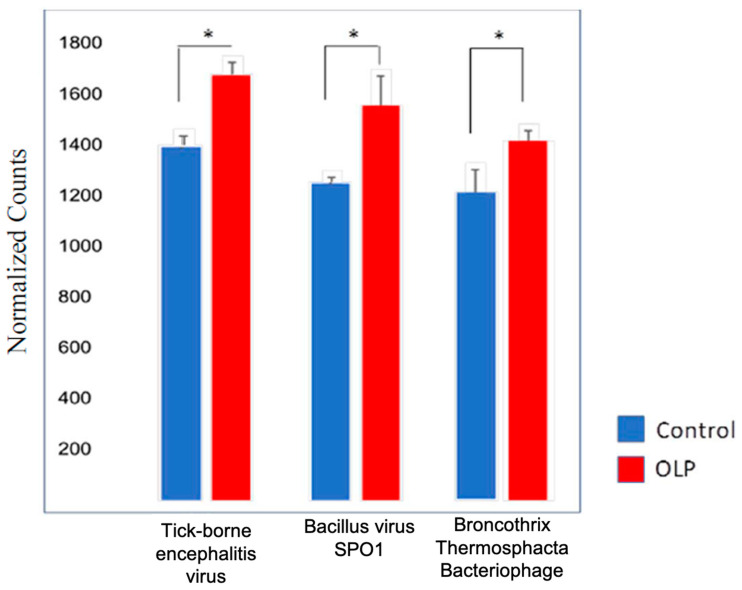
Three viral species are significantly different between OLP patients and healthy controls. P values are 0.0012, 0.0074, and 0. 036 respectively from left to right, error bars represent s.e.m. Virus counts for the majority of species identified were higher in OLP patients when compared to that of controls. * indicate statistical significance.

Considering a typical oral cavity contains more than 500 different bacterial species and various viruses and yeast [20], the oral microbiome may play a significant or yet undetermined role in OLP etiopathogenesis. Current technologies of next generation sequencing (NGS) provide a powerful tool to identify the potential pathogens of OLP and facilitate new potential therapies. Especially RNAseq which can simultaneously identify the microbial gene expression and the host gene expression. We performed molecular analysis with RNAseq in OLP patients as compared to healthy control patients (Table 1), and were able to identify putative candidates for OLP pathogenesis (Figure 3). Of the identified microorganisms (Table S2), 25 bacterial species were observed to be differentially present between OLP patients and healthy controls. As shown in Figure 3, although there is no unique bacterium associated with OLP samples, the abundance of bacteria species in the oral cavity is very different between OLP patients and healthy individuals. Comparing to healthy control, OLP samples have more Proteobacteria (54.7% vs. 39.0%), Fusobacteria (4.3% vs. 0.6%) and Spirochaetes (3.5% vs. 1.2%), but with less Actinobacteria (14.3% vs. 32.0%) and Firmicutes (9.7% vs. 15.0%). We further analyzed the abundancy of specific bacteria species in oral cavity between healthy individuals and OLP patients. With respect to bacterial species, higher prevalences of *Corynebacterium matruchotii*, *Fusobacterium periodonticum*, *Streptococcus intermedius*, *Streptococcus oralis* and *Prevotella denticola* are in patients with OLP as compared to controls (Figure 4). Clinically, these pathogens are significant, and in the context of OLP, it is established that patients with poor oral hygiene and plaque containing periodontopathogens respond poorly to conventional treatments for their OLP until their hygiene and plaque is adequately controlled. We also utilized PCR to confirm *Corynebacterium matruchotii*, *Fusobacterium periodonticum*, *Streptococcus intermedius*, *Streptococcus oralis* and *Prevotella denticola* are dominant bacteria in OLP patients versus controls (Figure 4). Finally, we also identified a higher abundance of viruses in OLP patients as compared to healthy controls. Three viral species were statistically significantly higher in OLP patients and controls (Figure 5), namely tick-borne encephalitis virus, bacillus virus SPO1, and a brochothrix bacteriophage virus.

## 4. Discussion

Since the causes of OLP have not been fully determined, there is no definitive cure for the condition. The first-line drug in the treatment of oral lichen planus is topical corticosteroids for their ability to modulate inflammation and immune responses by reducing the lymphocytic exudate and stabilizing the lysosomal membrane [21]. If lesions are erosive and painful, and topical steroids are not able to provide clinical relief and resolution or remission, then systemic corticosteroids may be administered [13,22]. Immune suppressants such as cyclosporine, a calcineurin inhibitor, are also used to reduce symptoms of inflammation and irritation [13]. Calcineurin is a protein phosphatase, which is involved in the activation of transcription of IL-2, and stimulates the growth and differentiation of the T-cell response. By using calcineurin inhibitors, the inflammation of T-cells, a suspected catalyst in the development of OLP, can be stopped [21].

However, without the underlying mechanism of OLP, current therapies are mainly empirical. In immunosuppressive therapy, topical calcineurin inhibitors (TCI) including tacrolimus, pimecrolimus, and cyclosporine are still controversial for use in this setting. Side effects, aside from oral candidiasis, are bad taste, nausea, dry mouth, sore throat, and swollen mouth but are considered minimal [23]. Topical retinoids such as tretinoin, isotretinoin, fenretinide, and tezarotene are generally less effective than topical corticosteroids and are more likely to cause adverse side effects [24]. When considering topical retinoids for treatment, positive effects of retinoids should be weighed against their rather significant side effects like cheilitis, elevation of serum liver enzymes and triglyceride levels, and teratogenicity [21]. Drugs like biologics or disease-modifying anti-rheumatic drugs (DMARDs) are also used in this setting, usually for cases that do not respond to steroid therapy.

Therefore, it is critical to identify molecular pathways active in oral cavity cells of OLP patients and the related pathogens to improve OLP treatment or resolution. Herein we found the HNF4A gene network activation in oral cavity cells of OLP patients, and several periodontopathogens, including *Prevotella denticola*, which dominated in patients with OLP. The hepatocyte nuclear factor-4-alpha (HNF4A) is a member of the nuclear receptor superfamily of ligand-dependent transcription factors [25] and is the most abundant DNA-binding protein in the liver, where it regulates genes largely involved in the hepatic gluconeogenic program and lipid metabolism [26]. Besides the liver and pancreas [27], it is highly expressed in the kidney and involved with drug metabolism [28], and also in the small intestine and colon where it is involved in inflammation [29,30]. HNF4A is also known to affect inflammation and immune pathways in other immune-mediated conditions such as Crohn’s disease [31] and inflammatory bowel syndrome [29].

The HNF4A network could explain previously elusive associations of OLP with various diseases. Previously chronic liver disease and hepatitis C have been found to be associated with OLP with the presence of HLA-DR 6 [32] and HLA-A3 alone [33]. Moreover, comorbidities or conditions like hypertension and diabetes mellitus have associations with lichen planus [13,34]. These associations maybe due to disease effects on the HNF4A gene network in modulating the inflammatory and immune pathways of cells in the oral cavity. HNF4A network activation also explains the association of OLP or OLP-like (lichenoid) reactions with specific pharmacotherapy. Examples of drugs or medications that have been associated with OLP or lichenoid drug reactions include beta blockers, nonsteroidal anti-inflammatory drugs, anti-malarials, diuretics, oral hypoglycemics, penicillamine, and oral retroviral medications [13]. These interesting associations of drug intake with OLP may be due to the systematic alteration of the HNF4A gene network and in the liver and kidney involving drug metabolism [28].

We also identified a potential major pathogen, *Prevotella denticola*, which could activate the HNF4A network in cells of the oral cavity. *Prevotella denticola* may serve as a target for future therapies in this context, though further and larger studies will be needed to more reliably assess and validate this association. *Prevotella* species are found in humans as opportunistic pathogens [35]. More than twenty identified species of *Prevotella* are known to cause infection [36]. *Prevotella denticola* is isolated from the human mouth, where it is suspected to cause disease [37], but its pathogenesis in OLP remains elusive until this study. Although *Prevotella denticola* is detected in samples from both healthy individual and OLP patients, the abundance of *Prevotella denticola* is higher in the oral cavity of OLP patients. It has been reported that increased *Prevotella* abundance is associated with augmented T-helper type 17 (Th17) mediated mucosal inflammation [38]. It has also been reported that HNF4A mutation is associated with Th17 cell-mediated inflamed gut mucosa [39,40]. Taken together, our findings support the hypothesis that *Prevotella denticola* abundance in the oral cavity can lead to activation of the HNF4A gene network in cells, resulting in Th17 mucosal inflammation and diseases like OLP.

We also found greater viral abundance in OLP patients as compared to healthy controls. Interestingly, OLP can have a clinical course similar to viral diseases affecting the oral cavity; specifically, the waxing and waning nature of the disease with bouts of active lesions and symptoms, followed by periods of quiescence or remission. Additionally, bacterial and viral interactions are key to many pathologic conditions, including those that involve the oral cavity. Certain bacteria have been shown to increase the lytic replication of oral herpesvirus pathogens and thus polymicrobial interactions are likely key to disease pathogenesis. We have previously characterized a significant number of bacteriophage viruses in oral infectious diseases such as osteomyelitis or osteonecrosis, and such profiles were more abundant in diseased patients versus healthy controls [41]. In this although a preliminary small-scale study, we found a higher abundance of bacteriophage in OLP patients as compared to controls. This study only includes a small population of OLP patients, and the results are not conclusive. The pathogenesis of OLP is complicated and more studies are needed, especially molecular analysis of the microbe populations in oral cavity of OLP patients. However, the findings reported here taken together suggest that bacterial and viral interactions are important to oral disease processes that have inflammatory components, and OLP may be another such disease modulated by complex microbial and host interactions. Our results represent an initial step of molecular analysis of OLP to facilitate and promote large scale molecular study of the OLP pathogenesis with more patients. Further studies with large number of OLP patients are required to confirm and apprehend the pathogenesis of OLP.

## Figures and Tables

**Table 1:** Clinicopathologic and demographic features of the study population cases and controls.

Cases	Age	Sex	Clinical Oral Diagnosis	Comorbidities	OLP Treatment
1	61	F	OLP (reticular) buccal mucosa bilateral and gingiva generalized	None	Dexamethasone oral elixir 0.5 mg/mL rinse and spit tid
2	76	M	OLP (erosive) buccal mucosa bilateral and gingiva generalized	Hypertension	Clobetasol gel 0.05% for topical use tid
3	62	F	OLP (reticular) buccal mucosa bilateral and gingival generalized	Hearing impairment	Fluocinonide gel 0.05% for topical use tid
4	63	F	OLP (erosive and reticular) buccal mucosa bilateral and gingiva generalized	Hypertension, angina	Fluocinonide gel 0.05% for topical use tid and dexamethasone as in case 1
5	61	F	OLP (reticular) buccal mucosa bilateral	Mitral valve prolapse, anemia	Dexamethasone as in case 1
6	63	F	OLP (erosive and reticular) buccal mucosa bilateral and tongue	Hypothyroidism, depression	Dexamethasone as in case 1
7	59	F	OLP (erosive and reticular) buccal mucosa bilateral and gingiva generalized	Anxiety	Dexamethasone as in case 1
8	72	F	OLP (erosive and reticular) buccal mucosa bilateral	Fibromyalgia, osteoarthritis, hypertension, hypothyroidism	Dexamethasone as in case 1
9	78	F	OLP (reticular) buccal mucosa bilateral and tongue	Hypertension, DMII	None
10	79	F	OLP (reticular) buccal mucosa bilateral	Hypothyroidism, osteoporosis	Dexamethasone as in case 1
Controls					
1	51	F	Simple bone cyst	None	N/A
2	69	F	Fractured dental filling	Hypertension	N/A
3	72	F	Psychogenic bruxism	None	N/A
4	59	M	Dental crown fracture	Hypertension	N/A
5	57	M	None	None	N/A

## Supplementary Materials

The following are available online at https://www.mdpi.com/2076-0817/9/11/952/s1, Table S1: Genes in human oral cells differentially expressed between OLP patients and healthy individuals, Table S2: Bacteria and virus detected with RNAseq in OLP patients and healthy individuals.

## References

[1] Baek K., Choi Y. (2018). The microbiology of oral lichen planus: Is microbial infection the cause of oral lichen planus?. Mol. Oral Microbiol..

[2] Di Stasio D., Guida A., Salerno C., Contaldo M., Esposito V., Laino L., Serpico R., Lucchese A. (2014). Oral lichen planus: A narrative review. Front. Biosci. Elite Ed..

[3] Axell T., Rundquist L. (1987). Oral lichen planus—A demographic study. Community Dent. Oral Epidemiol..

[4] Boñar-Alvarez P., Pérez-Sayáns M., Garcia-Garcia A., Chamorro-Petronacci C., Gándara-Vila P., Luces-González R., Rey E.O., Blanco-Carrión A., Suárez-Peñaranda J.M. (2019). Correlation between clinical and pathological features of oral lichen planus: A retrospective observational study. Med. Baltim..

[5] Olson M.A., Rogers R.S., Bruce A.J. (2016). Oral lichen planus. Clin. Dermatol..

[6] George S., Balan A. (2018). A potential side effect of oral topical steroids: Central serous chorioretinopathy. Indian J. Dent. Res..

[7] Carbone M., Goss E., Carrozzo M., Castellano S., Conrotto D., Broccoletti R., Gandolfo S. (2003). Systemic and topical corticosteroid treatment of oral lichen planus: A comparative study with long-term follow-up. J. Oral Pathol. Med..

[8] Bakshi S.S. (2017). A burning sensation in the mouth. Cleve. Clin. J. Med..

[9] Kim D., Pertea G., Trapnell C., Pimentel H., Kelley R., Salzberg S. (2013). TopHat2: Accurate alignment of transcriptomes in the presence of insertions, deletions and gene fusions. Genome Biol..

[10] Kramer A., Green J., Pollard J., Tugendreich S. (2014). Causal analysis approaches in Ingenuity Pathway Analysis. Bioinformatics.

[11] Wood D.E., Salzberg S.L. (2014). Kraken: Ultrafast metagenomic sequence classification using exact alignments. Genome Biol..

[12] Benson D.A., Cavanaugh M., Clark K., Karsch-Mizrachi I., Lipman D.J., Ostell J., Sayers E.W. (2013). GenBank. Nucleic Acids Res..

[13] Krupaa R.J., Sankari S.L., Masthan K.M.K., Rajesh E. (2015). Oral lichen planus: An overview. J. Pharm. Bioallied Sci..

[14] Hasan S. (2019). Lichen planus of lip—Report of a rare case with review of literature. J. Fam. Med. Prim. Care.

[15] Ismail S.B., Kumar S.K., Zain R.B. (2007). Oral lichen planus and lichenoid reactions: Etiopathogenesis, diagnosis, management and malignant transformation. J. Oral Sci..

[16] Van der Meij E.H., van der Waal I. (2003). Lack of clinicopathologic correlation in the diagnosis of oral lichen planus based on the presently available diagnostic criteria and suggestions for modifications. J. Oral Pathol. Med..

[17] Lehman J.S., Tollefson M.M., Gibson L.E. (2009). Lichen planus. Int. J. Dermatol..

[18] Tziotzios C., Lee J.Y., Brier T., Saito R., Hsu C.-K., Bhargava K., Stefanato C.M., Fenton D.A., McGrath J.A. (2018). Lichen planus and lichenoid dermatoses: Clinical overview and molecular basis. J. Am. Acad. Dermatol..

[19] Salehi B., Jornet P.L. (2019). Plant-Derived Bioactives in Oral Mucosal Lesions: A Key Emphasis to Curcumin, Lycopene, Chamomile, Aloe vera, Green Tea and Coffee Properties. Biomolecules.

[20] Moore W.E., Moore L.V. (2000). The bacteria of periodontal diseases. Periodontology.

[21] Lavanya N., Rao U.K., Jayanthi P., Ranganathan K. (2011). Oral lichen planus: An update on pathogenesis and treatment. J. Oral Maxillofac. Pathol..

[22] Alrashdan M.S., Cirillo N., McCullough M. (2016). Oral lichen planus: A literature review and update. Arch. Dermatol. Res..

[23] Thongprasom K., Dhanuthai K. (2008). Steriods in the treatment of lichen planus: A review. J. Oral Sci..

[24] Carrozzo M., Porter S., Mercadante V., Fedele S. (2019). Oral lichen planus: A disease or a spectrum of tissue reactions? Types, causes, diagnostic algorhythms, prognosis, management strategies. Periodontology 2000.

[25] Evans R.M., Mangelsdorf D.J. (2014). Nuclear Receptors, RXR, and the Big Bang. Cell.

[26] Chandra V., Huang P., Potluri N., Wu D., Kim Y., Rastinejad F. (2013). Multidomain integration in the structure of the HNF-4alpha nuclear receptor complex. Nature.

[27] Huang J., Levitsky L.L., Rhoads D.B. (2009). Novel P2 promoter-derived HNF4alpha isoforms with different N-terminus generated by alternate exon insertion. Exp. Cell Res..

[28] Martovetsky G., Tee J.B., Nigam S.K. (2013). Hepatocyte nuclear factors 4alpha and 1alpha regulate kidney developmental expression of drug-metabolizing enzymes and drug transporters. Mol. Pharmacol..

[29] Chahar S., Gandhi V., Yu S., Desai K., Cowper-Sal-lari R., Kim Y., Perekatt A.O., Kumar N., Thackray J.K., Musolf A. (2014). Chromatin profiling reveals regulatory network shifts and a protective role for hepatocyte nuclear factor 4alpha during colitis. Mol. Cell. Biol..

[30] Marcil V., Seidman E., Sinnett D., Boudreau F., Gendron F.-P., Beaulieu J.-F., Ménard D., Precourt L.-P., Amre D., Levy E. (2010). Modification in oxidative stress, inflammation, and lipoprotein assembly in response to hepatocyte nuclear factor 4alpha knockdown in intestinal epithelial cells. J. Biol. Chem..

[31] Marcil V., Sinnett D., Seidman E.G., Boudreau F., Gendron F.-P., Beaulieu J.-F., Menard D., Lambert M., Bitton A., Sanchez R. (2012). Association between genetic variants in the HNF4A gene and childhood-onset Crohn’s disease. Genes Immun..

[32] Carrozzo M., Brancatello F., Dametto E., Arduino P.G., Pentenero M., Rendine S., Porter S.R., Lodi G., Scully C., Gandolfo S. (2005). Hepatitis C virus-associated oral lichen planus: Is the geographical heterogeneity related to HLA-DR6?. J. Oral Pathol. Med..

[33] Lowe N.J., Cudworth A.G., Woodrow J.C. (1976). HL-A antigens in lichen planus. Br. J. Dermatol..

[34] Harries L.W., Locke J.M., Shields B., Hanley N., Hanley N., Steele A., Njølstad P.R., Ellard S., Hattersley A.T. (2008). The diabetic phenotype in HNF4A mutation carriers is moderated by the expression of HNF4A isoforms from the P1 promoter during fetal development. Diabetes.

[35] Alauzet C., Marchandin H., Lozniewski A. (2010). New insights into Prevotella diversity and medical microbiology. Future Microbiol..

[36] Ley R.E. (2016). Gut microbiota in 2015: Prevotella in the gut: Choose carefully. Nat. Rev. Gastroenterol. Hepatol..

[37] Ibrahim M., Subramanian A., Anishetty S. (2017). Comparative pan genome analysis of oral Prevotella species implicated in periodontitis. Funct. Integr. Genom..

[38] Larsen J.M. (2017). The immune response to Prevotella bacteria in chronic inflammatory disease. Immunology.

[39] Paul G., Khare V., Gasche C. (2012). Inflamed gut mucosa: Downstream of interleukin-10. Eur. J. Clin. Investig..

[40] Barrett J.C., Lee J.C., Lees C.W., Prescott N.J., Anderson C.A., Phillips A., Wesley E., Parnell K., Zhang H., Drummond H. (2009). Genome-wide association study of ulcerative colitis identifies three new susceptibility loci, including the HNF4A region. Nat. Genet..

[41] Sedghizadeh P.P., Yooseph S., Fadrosh D.W., Zeigler-Allen L., Thiagarajan M., Salek H., Farahnik F., Williamson S.J. (2012). Metagenomic investigation of microbes and viruses in patients with jaw osteonecrosis associated with bisphosphonate therapy. Oral Surg. Oral Med. Oral Pathol. Oral Radiol..

