# Supplementary material for: Genomic Analysis of Oral Lichen Planus and Related Oral Microbiome Pathogens
Source: Pathogens. 2020 Nov 16;9(11):952. doi: 10.3390/pathogens9110952 (PMC7697643; doi:10.3390/pathogens9110952)
Supplement: Supplementary file 1 [file pathogens-09-00952-s001.pdf]

Table S2. Bacteria and virus detected with R	
OLP	
Microbiome Species	Average Counts (normalized)
Veillonella parvula	3435527.229
Rothia mucilaginosa	1810713.571
Haemophilus parainfluenzae	844236.8342
Fusobacterium nucleatum	825289.7789
Neisseria meningitidis	626843.5897
Achromobacter xylosoxidans	415495.0883
Atopobium parvulum	205918.2297
Campylobacter concisus	159293.9124
Leptotrichia buccalis	123966.9359
Megasphaera elsdenii	87368.48455
Prevotella melaninogenica	82285.23784
Selenomonas sputigena	77508.6755
Haemophilus influenzae	76896.39289
Porphyromonas gingivalis	75766.09645
Rothia dentocariosa	64620.85367
Candidatus Saccharimonas aalborgensis	61728.68147
Aggregatibacter aphrophilus	54899.61834
Prevotella intermedia	37434.48581
Tannerella forsythia	36640.47285
Streptococcus parasanguinis	34865.49274
Selenomonas ruminantium	32825.83925
Streptococcus pneumoniae	23422.9219
Pseudogulbenkiania sp. NH8B	23371.8297
Neisseria lactamica	21815.23198
Streptococcus constellatus	20678.39506
Streptococcus pyogenes	20154.71044
Dichelobacter nodosus	19653.086
Prevotella sp. oral taxon 299	19244.10773
Capnocytophaga ochracea	18866.69759
[Eubacterium] eligens	17926.74096
Streptococcus mitis	17758.73348
Campylobacter curvus	17565.59393
Taylorella equigenitalis	15652.75392
Candidatus Saccharibacteria bacterium RAAC3_TM7_1	15478.8893
Streptococcus oligofermentans	15445.0097
Ruminiclostridium thermocellum	15128.26924
Kocuria rhizophila	14534.55059
[Clostridium] saccharolyticum	13834.76647
Mobiluncus curtisii	12226.83711
Porphyromonas asaccharolytica	11934.89197
Alteromonas macleodii	11795.64654
Campylobacter jejuni	11434.08098
Alkaliphilus metalliredigens	11110.06602
Clostridium sp. SY8519	10660.96776
Candidatus Kinetoplastibacterium galatii	10306.05213
Streptococcus dysgalactiae	9921.069259
Beutenbergia cavernae	9847.025451
Mannheimia haemolytica	9318.389064
Escherichia coli	9075.501643
Streptococcus sanguinis	8883.458496

Candidatus Hamiltonella defensa	8765.999689
Clostridium botulinum	8618.283278
Propionibacterium avidum	8480.487263
Neisseria gonorrhoeae	8403.95921
Eubacterium rectale	8321.721258
Jonesia denitrificans	8130.988625
Hyphomicrobium nitrativorans	7663.281185
Campylobacter hominis	7573.253666
Prevotella dentalis	7133.078687
Mycoplasma hyopneumoniae	6906.389526
Corynebacterium argentoratense	6558.384485
Thauera sp. MZ1T	6521.481732
[Clostridium] stercorarium	6036.080454
Leadbetterella byssophila	5745.60771
Streptococcus salivarius	5084.465299
Arcanobacterium haemolyticum	4926.291111
Macrococcus caseolyticus	4869.007226
Micrococcus luteus	4834.201935
Streptococcus equi	4825.310544
Oscillibacter valericigenes	4679.309626
butyrate-producing bacterium SSC/2	4635.132016
Ilyobacter polytropus	4632.362662
Roseburia hominis	4605.968475
Coprococcus catus	4513.087167
Campylobacter fetus	4486.93473
Megamonas hypermegale	4231.847878
Riemerella anatipestifer	4217.218421
Bacillus thuringiensis	4070.825111
Thiocystis violascens	4049.011888
Lachnoclostridium phytofermentans	4014.829704
Carnobacterium maltaromaticum	3630.422343
Sebaldella termitidis	3476.406887
Staphylococcus haemolyticus	3307.959463
Syntrophobacter fumaroxidans	3271.401975
Clostridium kluyveri	3258.260924
Filifactor alocis	3025.027123
Streptococcus sp. I-P16	2997.089729
Streptococcus pseudopneumoniae	2972.717776
Bacteroides vulgatus	2928.198821
[Mannheimia] succiniciproducens	2907.150286
Collimonas fungivorans	2819.838416
Magnetococcus marinus	2731.524626
Thiobacillus denitrificans	2730.319277
Prevotella ruminicola	2716.013351
Streptococcus sp. I-G2	2699.422993
Niastella koreensis	2639.941347
Ralstonia solanacearum	2635.626152
Streptococcus gordonii	2632.496209
Paludibacter propionicigenes	2570.068778
Jingmen tick virus	2530.66705
Comamonas testosteroni	2482.093745
Peptoclostridium difficile	2452.189537
Lactobacillus fermentum	2392.363769

Azoarcus sp. BH72	2303.878671
Flavobacteriaceae bacterium 3519-10	2222.428093
Streptococcus anginosus	2183.583062
Butyrivibrio proteoclasticus	2165.709168
[Eubacterium] siraeum	2054.380865
Kribbella flavida	2029.937342
Cryptobacterium curtum	1992.232708
Candidatus Zinderia insecticola	1974.882081
Parabacteroides distasonis	1826.54615
Anaerobaculum mobile	1822.778671
Olsenella uli	1802.860078
Halanaerobium hydrogeniformans	1784.372651
Tick-borne encephalitis virus	1747.517785
Saccharopolyspora erythraea	1728.572881
Rhodospirillum rubrum	1688.637738
Staphylococcus epidermidis	1678.594116
Sodalis glossinidius	1627.345766
Erysipelothrix rhusiopathiae	1591.419709
Bacillus virus SPO1	1583.029071
Anaplasma centrale	1519.426383
Treponema denticola	1473.378508
Capnocytophaga canimorsus	1458.052508
Streptococcus macedonicus	1456.423232
Aggregatibacter actinomycetemcomitans	1438.230003
Anaerococcus prevotii	1423.209805
Cellulosilyticum lentocellum	1421.651868
Brachybacterium faecium	1414.977586
Bronchotrix virus Thermophacta bacteriphage	1394.254104
Actinobacillus succinogenes	1373.762371
Thioalkalivibrio sulfidiphilus	1369.544862
Thioalkalimicrobium cyclicum	1364.703406
Mycobacterium sp. JDM601	1363.586593
Tetragenococcus halophilus	1363.553446
Mesotoga prima	1362.781572
Candidatus Puniceispirillum marinum	1309.635311
Leptothrix cholodnii	1307.452571
Eubacterium limosum	1305.74138
Gramella forsetii	1286.069822
Thermanaerovibrio acidaminovorans	1283.739927
candidate division SR1 bacterium RAAC1_SR1_1	1245.131342
Pasteurella multocida	1219.235619
Flavobacterium psychrophilum	1213.840017
Nocardiopsis alba	1199.073393
Halothiobacillus neapolitanus	1178.684166
Candidatus Carsonella ruddii	1168.090398
Thermosediminibacter oceani	1123.529705
Mycoplasma hyorhinis	1118.291558
Lactobacillus reuteri	1086.991491
Alistipes shahii	1081.1798
Frateuria aurantia	1050.701639
Acidothermus cellulolyticus	1034.362384
Candidatus Profftella armatura	1030.539615
Desulfomonile tiedjei	1012.588737

Streptococcus suis	1004.021005
Frankia sp. EuI1c	966.7181843
Mahella australiensis	966.5100106
Pseudonocardia dioxanivorans	960.2329669
Owenweeksia hongkongensis	959.987509
Nocardia farcinica	957.8158926
Finegoldia magna	953.2863447
Clostridium acetobutylicum	943.5274921
Haemophilus parasuis	894.3916004
Pseudomonas aeruginosa	879.65122
Dickeya dadantii	875.1803446
Rickettsia australis	870.5931162
Corynebacterium urealyticum	843.3037006
Propionibacterium acnes	819.2572046
Actinobacillus pleuropneumoniae	797.9658716
Caldicellulosiruptor kronotskyensis	781.2828121
Faecalitalea cylindroides	776.1484477
Corynebacterium diphtheriae	767.4445618
Pedobacter heparinus	748.6248488
Kineococcus radiotolerans	732.0870205
Chitinophaga pinensis	731.5443735
Amycolicicoccus subflavus	731.0443786
Histophilus somni	729.6492699
Methylomicrobium alcaliphilum	728.0514733
Oceanithermus profundus	703.6491702
Thermovirga lienii	700.7230242
Thermobispora bispora	690.8909457
Streptococcus uberis	687.3719373
Bacillus coagulans	626.0015219
Bacillus cytotoxicus	618.4377566
Dill cryptic virus 2	575.8866491
Haemophilus ducreyi	571.784698
Bifidobacterium longum	571.4486826
Marivirga tractuosa	565.7122803
Coprococcus sp. ART55/1	546.5359034
Desulfotomaculum gibsoniae	526.4780525
Spring beauty latent virus	522.6202009
alpha proteobacterium HIMB59	493.025217
Aminobacterium colombiense	492.8863417
Buchnera aphidicola	492.5961806
Clostridium tetani	492.1856025
Burkholderia multivorans	480.8704346
Corynebacterium kroppenstedtii	475.6220403
Gallibacterium anatis	474.2792235
Lactobacillus sanfranciscensis	463.9387069
Solitalea canadensis	462.0094581
Actinobacillus suis	444.8284605
Stenotrophomonas maltophilia	439.7124614
Flavobacterium indicum	425.2601622
Methanococcus maripaludis	414.6080959
Planctomyces limnophilus	413.2721691
Streptococcus agalactiae	411.2400624
Aequorivita sublithincola	407.4323932

Bibersteinia trehalosi	402.3423612
Desulfotomaculum reducens	402.0242253
Pedobacter saltans	401.4698498
Mouse astrovirus M-52/USA/2008	397.7216543
Amphibacillus xylanus	394.2993765
Leptospirillum ferriphilum	391.7936064
Caldilinea aerophila	387.7081532
Mycobacterium rhodesiae	385.2670847
Candidatus Azobacteroides pseudotrichonymphae	384.6312384
Segniliparus rotundus	383.9315467
Lactobacillus kefiranofaciens	373.273856
Corynebacterium efficiens	372.7355969
Propionibacterium propionicum	360.1445988
Aerococcus urinae	346.0534359
[Ruminococcus] torques	340.0362423
Chromobacterium violaceum	336.7411534
Meiothermus silvanus	334.5053257
Ethanoligenens harbinense	327.8403629
Burkholderia gladioli	311.4201316
Streptococcus thermophilus	306.9012228
Streptococcus mutans	304.75375
Sulfobacillus acidophilus	304.5510708
Prochlorococcus marinus	300.6024959
Brevibacillus brevis	299.3059003
[Clostridium] sticklandii	299.2999382
Ramlibacter tataouinensis	286.6296702
Geitlerinema sp. PCC 7407	275.9327419
Candidatus Baumannia cicadellinicola	270.8723685
Lactobacillus salivarius	259.8376415
Bacteroides thetaiotaomicron	251.1598996
Fibrobacter succinogenes	243.2188239
Clostridium beijerinckii	240.4406627
Desulfobulbus propionicus	236.4897488
Melioribacter roseus	235.2160049
Candidatus Sulcia muelleri	232.5400588
Lactococcus lactis	229.7529346
Desulfitobacterium dichloroeliminans	226.7415423
Deferribacter desulfuricans	226.6754987
Flexistipes sinusarabici	222.7051558
Fretibacterium fastidiosum	218.9041818
Cyanothece sp. PCC 7425	215.094391
Enterococcus hirae	211.2618928
Odoribacter splanchnicus	206.107572
Hop trefoil cryptic virus 2	198.8389921
Bacteroides helcogenes	191.4084936
Caldanaerobacter subterraneus	188.4816382
Variovorax paradoxus	184.5361171
Salinibacter ruber	183.3709925
Salmonella enterica	181.6727863
Streptococcus iniae	175.9874943
Streptococcus lutetiensis	170.6441875
Bifidobacterium dentium	170.4209654
Gottschalkia acidurici	169.1478881

Streptobacillus moniliformis	167.5136491
Caldicellulosiruptor saccharolyticus	167.1513518
Bacteroides salanitronis	166.1361272
Staphylococcus lugdunensis	165.3949863
Flavobacterium branchiophilum	164.9795465
Desulfotalea psychrophila	164.9723789
Lactococcus garvieae	164.5672852
Bordetella avium	162.7852976
Acetobacterium woodii	158.8352629
Helicobacter cinaedi	158.157163
Belliella baltica	157.484672
Truepera radiovictrix	153.5305036
Rubrobacter xylanophilus	138.910921
Vibrio cholerae	137.8634698
Bifidobacterium thermophilum	136.8786984
Torque teno midi virus 2	135.5230165
Melissococcus plutonius	134.3574369
Streptococcus parauberis	133.86099
Thermomonospora curvata	131.9862537
Treponema brennaborense	129.2556459
Desulfobacterium autotrophicum	127.3040671
Myxococcus fulvus	126.2358128
Hepatitis C virus	125.66876
Burkholderia phymatum	125.5171709
Ruminococcus sp. SR1/5	124.9497906
Desulfomicrobium baculatum	124.4120991
Acholeplasma palmae	124.3165013
Clostridiales genomosp. BVAB3	120.2948912
Yersinia enterocolitica	119.8420326
Candidatus Methylomirabilis oxyfera	118.9805623
Mycobacterium kansasii	118.8631273
Burkholderia glumae	116.9879938
Corynebacterium matruchotii	112.5840342
Bacillus subtilis	111.2612911
secondary endosymbiont of Ctenarytaina eucalypti	110.4537671
Staphylococcus aureus	109.0117339
Nitrosomonas eutropha	108.3726245
Weissella koreensis	107.6672062
Ochrobactrum anthropi	102.7081569
Cyclobacterium marinum	102.0108255
Prevotela denticola	98.55122261
Staphylococcus warneri	97.55269569
Providencia stuartii	96.48371994
Streptococcus infantarius	95.02194504
Enterococcus faecium	93.84679666
Rubrivivax gelatinosus	92.35236685
Serratia symbiotica	92.0715124
Streptococcus oralis	91.08160178
Vibrio campbellii	89.40923667
Enterobacter cloacae	87.14171579
Cyanobacterium stanieri	87.13052036
Lysinibacillus sphaericus	86.50284441
Acidaminococcus intestini	85.6184324

Blattabacterium sp. (Blatta orientalis)	84.80538898
Rhodanobacter denitrificans	83.52970829
Oceanobacillus iheyensis	82.94666715
Streptomyces pratensis	82.51426695
Corynebacterium aurimucosum	82.08249968
Bacillus cellulosilyticus	82.08062523
Mycoplasma hominis	81.37873682
Alicycliphilus denitrificans	81.29110825
Intrasporangium calvum	80.27472623
Tolumonas auensis	80.14292475
Lactobacillus acidophilus	79.72947449
Bifidobacterium asteroides	78.22586066
Listeria monocytogenes	76.40008282
Pandoravirus dulcis	75.81134905
Muricauda ruestringensis	74.68164645
Tepidanaerobacter acetatoxydans	74.29831554
Klebsiella variicola	73.62994109
Methylophaga nitratireducenticrescens	73.30048414
Pantoea sp. At-9b	73.04304307
Sphingobacterium sp. 21	72.93913018
Tsukamurella paurometabola	72.76696694
Roseobacter denitrificans	71.72653884
Candidatus Symbiobacter mobilis	71.1787151
Alkalilimnicola ehrlichii	70.88686985
Fusobacterium periodonticum	69.66839077
Acidaminococcus fermentans	69.57149148
Propionibacterium freudenreichii	68.88752597
Microbacterium testaceum	68.88383988
Primula malacoides virus 1	68.83020643
Treponema pedis	67.96804325
Acholeplasma laidlawii	67.96092371
Wigglesworthia glossinidia	67.56083931
Desulfurispirillum indicum	67.11990726
Bifidobacterium adolescentis	66.5680607
Pseudoalteromonas haloplanktis	66.4149126
Nitratiruptor sp. SB155-2	66.1879491
Bacillus cereus	65.88059654
Renibacterium salmoninarum	65.85783533
Catenulispora acidiphila	65.43531446
Geobacter lovleyi	63.55664894
Bacillus amyloliquefaciens	63.50621069
Verminephrobacter eiseniae	63.39580371
Dickeya phage RC-2014	63.10131901
Clostridium perfringens	63.01226352
Photobacterium profundum	62.98493554
Treponema succinifaciens	62.78341606
[Ruminococcus] obeum	62.73724374
Marinomonas posidonica	61.3602584
Moorella thermoacetica	60.15338866
Xanthomonas campestris	60.00068809
Deinococcus proteolyticus	59.85067033
Aliivibrio fischeri	59.38300452
Oenococcus oeni	59.24858769

Carnobacterium sp. 17-4	57.52873153
Psychroflexus torquis	57.46979991
Desulfarculus baarsii	57.44141558
Carnobacterium sp. WN1359	56.45910101
Thioalkalivibrio sp. K90mix	56.16450549
Halorhodospira halophila	53.65843124
Slackia heliotrinireducens	53.10803549
Psychromonas sp. CNPT3	52.87625745
Dechloromonas aromatica	52.8511068
Candidatus Protochlamydia amoebophila	52.42872085
Halobacillus halophilus	52.16557256
Red clover cryptic virus 2	52.13340106
Opitutus terrae	52.09806573
Coxiella burnetii	51.62318416
Salivirus FHB	51.48229735
Desulfovibrio salexigens	50.71531769
Arthrobacter arilaitensis	50.67201597
Syntrophomonas wolfei	50.38968174
Ferrimonas balearica	49.71999605
Bacteroides fragilis	49.709828
Lactobacillus sakei	49.39686465
Ammonifex degensii	48.9043539
Pseudomonas mendocina	48.63385755
Shewanella baltica	48.61775862
Shigella flexneri	48.50075196
Pyrolobus fumarii	47.59970679
Novosphingobium aromaticivorans	46.55524129
Oceanimonas sp. GK1	46.36069692
Pseudomonas stutzeri	45.66637557
Butyrivibrio fibrisolvens	45.65466515
Leuconostoc citreum	45.4709675
Kyrpidia tusciae	44.76671861
Mycobacterium smegmatis	44.38886153
Vibrio vulnificus	43.72894923
Geobacillus sp. GHH01	43.20378822
Candidatus Tremblaya princeps	43.07068263
Thermodesulfatator indicus	42.49077183
Rhodothermus marinus	42.21754931
Helicobacter pylori	42.13276462
Candidatus Solibacter usitatus	41.84959305
Methylobacterium extorquens	41.58846589
Ilumatobacter coccineus	41.1773546
Enterococcus casseliflavus	40.84256099
Malvastrum leaf curl Philippines betasatellite	40.58878676
Exiguobacterium sp. AT1b	40.52056778
Lawsonia intracellularis	40.34763466
Oscillatoria nigro-viridis	39.96623818
Leuconostoc carnosum	39.57684147
Stackebrandtia nassauensis	39.3041147
Legionella longbeachae	39.12793816
Synechococcus sp. RCC307	39.02452102
Tropheryma whipplei	38.67689114
Enterococcus mundtii	38.60183648

Streptococcus gallolyticus	38.51156357
Colwellia psychrerythraea	38.29746758
Desulfotomaculum acetoxidans	38.17164745
alpha proteobacterium HIMB5	37.83066321
Leptospira borgpetersenii	37.55527287
Streptococcus pasteurianus	37.39899216
Xylanimonas cellulosilytica	37.37155337
Ruminococcus bromii	37.30597385
Clostridium cellulovorans	37.12956392
Streptosporangium roseum	37.07927497
Bordetella petrii	37.02546586
Caldicellulosiruptor obsidiansis	37.00537513
[Clostridium] clariflavum	36.96970307
Alicyclobacillus acidocaldarius	36.84645982
Corynebacterium glutamicum	36.83179731
Spirochaeta smaragdinae	36.80561929
Desulfotomaculum ruminis	36.67677962
Clostridium pasteurianum	36.3612394
Gordonia polyisoprenivorans	36.1266511
Rhodoferax ferrireducens	35.95716095
Pectobacterium carotovorum	35.51025241
Desulfovibrio vulgaris	35.22453034
Corynebacterium maris	35.19668511
Bacillus pseudofirmus	35.1033011
Proteus mirabilis	35.09170635
Photorhabdus asymbiotica	35.01892546
Halothermothrix orenii	34.87833463
Streptomyces bingchenggensis	34.84710155
Anoxybacillus flavithermus	34.75180947
Burkholderia pseudomallei	34.5152049
Candidatus Endolissoclinum faulkneri	34.16397077
Chthonomonas calidirosea	33.62989297
Arcobacter butzleri	33.07911714
Haliangium ochraceum	32.82714017
Bacillus megaterium	32.35229709
Candidatus Riesia pediculicola	32.3040003
Rhodopseudomonas palustris	32.11747054
Syntrophothermus lipocalidus	32.10214149
Pseudoalteromonas atlantica	32.05223747
Eggerthella sp. YY7918	32.02089133
Lactobacillus delbrueckii	31.9745867
Acinetobacter oleivorans	31.84608862
Rickettsia canadensis	31.74749801
Paenibacillus polymyxa	31.62905492
Burkholderia mallei	31.57522657
Treponema primitia	31.44522722
Teredinibacter turnerae	31.36955423
Laribacter hongkongensis	31.25153943
Spirochaeta thermophila	30.67589457
Aliivibrio salmonicida	30.47564784
Vibrio furnissii	30.32601503
Kosmotoga olearia	30.21292784
Shimwellia blattae	29.97456675

Faecalibacterium prausnitzii	29.810909
Methanococcus aeolicus	29.58663058
Mycoplasma arthritidis	29.58079591
Vibrio anguillarum	29.10085443
Bacteriovorax marinus	28.61591075
Delftia acidovorans	28.53863054
Listeria welshimeri	27.9704227
Clostridium novyi	27.69104803
Dill cryptic virus 1	27.67327928
Thermodesulfobium narugense	27.58885542
Klebsiella oxytoca	27.41763547
Marinomonas sp. MWYL1	27.30017641
Psychromonas ingrahamii	27.119349
Saccharomonospora viridis	26.9265178
Leifsonia xyli	26.88701036
Mycoplasma putrefaciens	26.88229203
Carp picornavirus 1	26.86499493
Mycobacterium canettii	26.85215379
Bartonella grahamii	26.71653618
Ralstonia pickettii	26.58366884
Clavibacter michiganensis	26.38307566
Methylobacterium radiotolerans	26.27095812
Burkholderia phytofirmans	26.07319444
Sphaerochaeta globosa	26.0363216
Weeksella virosa	25.99576268
Burkholderia thailandensis	25.67181804
Pediococcus claussenii	25.64023851
Ruminococcus champanellensis	25.52829177
Advenella kashmirensis	25.40742823
Geobacter bemidjiensis	24.82074435
Lactobacillus gasseri	24.73539906
Cellvibrio japonicus	24.46177235
Brachyspira murdochii	24.45304559
Bifidobacterium breve	24.43728825
Burkholderia cenocepacia	24.3979516
Clostridium saccharobutylicum	24.30355946
Candidatus Liberibacter solanacearum	24.2499886
Sanguibacter keddieii	24.09259381
Serratia marcescens	24.08198309
Photorhabdus luminescens	24.0363209
Cyprinid herpesvirus 3	23.95213529
Halobacteroides halobius	23.87388543
Spirochaeta africana	23.77495062
Acholeplasma brassicae	23.65271325
Kinetoplastibacterium blastocrithidii	23.36197948
Thermoanaerobacter wiegelii	23.23661175
Anaerolinea thermophila	23.09985856
Staphylococcus carnosus	23.08328799
Isosphaera pallida	22.92653563
Human endogenous retrovirus K	22.64849145
Methylococcus capsulatus	22.557552
Thermobacillus composti	22.32924103
Candidatus Hodgkinia cicadicola	22.31826461

Rickettsia africae	22.26565854
Paenibacillus sp. Y412MC10	22.25470136
Nostoc punctiforme	22.21407985
Campylobacter coli	22.13582998
Kangiella koreensis	22.10731821
Coriobacterium glomerans	22.09160422
Candidatus Tremblaya phenacola	22.06223586
butyrate-producing bacterium SS3/4	21.81145706
Cyprinid herpesvirus 1	21.72890278
Pediococcus pentosaceus	21.54749553
Methylophaga frappieri	21.37239459
Heliobacterium modesticaldum	21.29573271
Kytococcus sedentarius	21.29408214
Leuconostoc mesenteroides	20.89617768
Candidatus Uzinura diaspidicola	20.89004703
Dictyoglomus thermophilum	20.87576947
Solibacillus silvestris	20.8439999
Corynebacterium jeikeium	20.6988664
Propionibacterium acidipropionici	20.53555511
Desulfurivibrio alkaliphilus	20.49006371
Pandoravirus salinus	20.40793772
Candidatus Sulfuricurvum sp. RIFRC-1	20.38771955
[Cellvibrio] gilvus	20.07158024
Pirellula staleyi	20.0626201
Flexibacter litoralis	20.06081797
Marinitoga piezophila	19.98921601
Caldicellulosiruptor bescii	19.92807808
Petrotoga mobilis	19.78823789
Alkaliphilus oremlandii	19.70692513
Burkholderia ambifaria	19.62880758
Burkholderia sp. KJ006	19.58558995
Mesoplasma florum	19.50429644
Pseudomonas denitrificans	19.44152718
Adlercreutzia equolifaciens	19.32653679
Listeria ivanovii	19.2715054
Syntrophobotulus glycolicus	19.05091306
Thermodesulfobacterium geofontis	18.91083948
Herminiimonas arsenicoxydans	18.85152292
Moraxella catarrhalis	18.76489275
Polaribacter sp. MED152	18.66072722
Gardnerella vaginalis	18.57857713
Nocardiopsis dassonvillei	18.55085934
Chroococcidiopsis thermalis	18.37691322
Mycoplasma penetrans	18.33862807
Sideroxydans lithotrophicus	18.26809683
Bacillus sp. 1NLA3E	18.19718065
Marinobacter hydrocarbonoclasticus	18.12562204
Cellulomonas flavigena	17.82245131
Shewanella loihica	17.76990784
Vibrio parahaemolyticus	17.73522206
Gordonia sp. KTR9	17.73052784
Pseudanabaena sp. PCC 7367	17.6330487
Mycobacterium abscessus	17.61965183

Pusillimonas sp. T7-7	17.4037537
Candidatus Ruthia magnifica	17.37481364
Actinoplanes friuliensis	17.34175433
Legionella pneumophila	17.31120704
Idiomarina loihiensis	17.26350933
Methylobacterium populi	17.1778873
Gallionella capsiferriformans	17.08497493
Acinetobacter calcoaceticus	17.07597143
Spirosoma linguale	16.83481933
Lactobacillus crispatus	16.77653014
Corynebacterium pseudotuberculosis	16.47867683
Pseudomonas putida	16.47318862
Paenibacillus terrae	16.33045634
Marinithermus hydrothermalis	16.25994921
Glaciecola psychrophila	16.22121165
Bradyrhizobium sp. BTAi1	16.1156097
Arthrobacter phenanthrenivorans	16.03446774
Chromohalobacter salexigens	16.01266646
Thermoanaerobacterium thermosaccharolyticum	16.00942791
Flavobacterium columnare	15.99515034
Candidatus Rickettsia amblyommii	15.94213522
Janthinobacterium sp. Marseille	15.84307297
Herbaspirillum seropedicae	15.80412126
Dokdonia sp. 4H-3-7-5	15.65937271
Pleurocapsa minor	15.4129273
Lactobacillus brevis	15.37851826
Magnetospirillum gryphiswaldense	15.37830412
Bifidobacterium bifidum	15.30061486
Pseudomonas fluorescens	15.2374173
Gluconobacter oxydans	14.96755852
Klebsiella pneumoniae	14.83072349
Enterococcus faecalis	14.79114859
Starkeya novella	14.75549804
Clostridium sp. BNL1100	14.66683236
Elusimicrobium minutum	14.4499261
Rhodococcus erythropolis	14.40793075
Halanaerobium praevalens	14.21591278
Mycoplasma mobile	14.00758173
Candidatus Blochmannia floridanus	13.99718515
Falconid herpesvirus 1	13.90787704
Thermincola potens	13.90298791
Burkholderia lata	13.84101264
Herpetosiphon aurantiacus	13.71079915
Cellulomonas fimi	13.65570516
Symbiobacterium thermophilum	13.58457484
Delftia sp. Cs1-4	13.58232518
Francisella tularensis	13.45899297
Microlunatus phosphovorus	13.44145761
Rhodococcus pyridinivorans	13.42004127
Nitrosomonas sp. Is79A3	13.24984869
Hydrogenobaculum sp. HO	13.14180218
Thermotoga neapolitana	13.12000089
Hippea maritima	12.94897097

Glaciecola nitratireducens	12.90879699
Corynebacterium halotolerans	12.90165821
Ruminococcus albus	12.8233891
Corynebacterium resistens	12.77772691
Lactobacillus johnsonii	12.65806154
Bacillus pumilus	12.63503797
Arthrobacter chlorophenolicus	12.60466148
Burkholderia rhizoxinica	12.46501619
Shewanella piezotolerans	12.26626849
Streptomyces venezuelae	12.25769328
Spiroplasma chrysopicola	12.23383237
Cupriavidus metallidurans	12.21181694
Candidatus Portiera aleyrodidarum	12.19449573
Paenibacillus sp. JDR-2	12.19082889
Methanosarcina mazei	12.00593045
Dehalogenimonas lykanthroporepellens	11.99656125
Acinetobacter baumannii	11.96618476
Treponema azotonutricium	11.90097094
secondary endosymbiont of Heteropsylla cubana	11.88748736
Cellulophaga algicola	11.7339928
Geobacillus thermoglucosidasius	11.71096924
Shewanella sp. ANA-3	11.66753746
Arthrobacter sp. FB24	11.53951103
[Clostridium] cellulolyticum	11.46550785
Plautia stali symbiont	11.4565477
Fibrella aestuarina	11.34588584
Rhodospirillum photometricum	11.17809447
uncultured Termite group 1 bacterium	11.11223821
Desulfovibrio piezophilus	11.01559641
Staphylococcus saprophyticus	10.99278699
Thermoanaerobacterium xylanolyticum	10.96832699
Streptococcus phage 5093	10.94487514
Calditerrivibrio nitroreducens	10.7204644
Acidimicrobium ferrooxidans	10.70127847
Fervidobacterium nodosum	10.69557611
Burkholderia cepacia	10.58262124
Clostridium saccharoperbutylacetonicum	10.4974275
Mycoplasma parvum	10.44014645
Enterobacter sp. 638	10.42953572
Synechococcus sp. PCC 7502	10.4277336
Persephonella marina	10.42016653
Formica exsecta virus 1	10.40955581
Xenorhabdus bovienii	10.3887819
Acetohalobium arabaticum	10.38612319
Desulfosporosinus acidiphilus	10.38303619
Bacillus infantis	10.27055296
Thiomicrospira crunogena	10.25258931
Bradyrhizobium diazoefficiens	10.21262947
Helicobacter bizzozeronii	10.20874848
Bacillus weihenstephanensis	10.20426841
Frankia symbiont of Datisca glomerata	10.17410606
Sphaerobacter thermophilus	10.14027687
Rahnella aquatilis	10.12274151

Psychrobacter sp. PRwf-1	10.11682501
Coprothermobacter proteolyticus	10.07463475
Mycoplasma fermentans	10.01000078
Nakamurella multipartita	9.78882859
Acidithiobacillus ferrivorans	9.691801847
Brucella melitensis	9.65306429
Azospirillum brasilense	9.651627863
Francisella sp. TX077308	9.593124533
Citrobacter koseri	9.56988682
Pantoea ananatis	9.556208343
Candidatus Phytoplasma mali	9.523002346
White clover cryptic virus 2	9.445741377
Geobacter uraniireducens	9.364813565
Sulfuricella denitrificans	9.249243331
Komagataeibacter medellinensis	9.243497624
Methanospirillum hungatei	9.178692852
Hirschia baltica	9.137724879
Nitrosomonas sp. AL212	9.079007402
Pepino mosaic virus	9.068610827
Thermobifida fusca	8.994821797
Cellulophaga lytica	8.77322132
Maribacter sp. HTCC2170	8.680888798
Xylella fastidiosa	8.623821894
Treponema caldaria	8.408352064
Rickettsia bellii	8.403828639
Streptomyces avermitilis	8.37366629
Elephantid herpesvirus 1	8.299064028
Mycoplasma pulmonis	8.273381754
Nocardioides sp. JS614	8.270723046
Candidatus Cloacimonas acidaminovorans	8.258096055
Mycobacterium leprae	8.186751587
Paenibacillus mucilaginosus	8.182442308
Ornithobacterium rhinotracheale	8.10091541
Bradyrhizobium japonicum	8.051995423
Halyomorpha halys symbiont	7.993492092
Eggerthella lenta	7.976769967
Kitasatospora setae	7.966373391
Cardinium endosymbiont of Encarsia pergandiella	7.94394891
Bradyrhizobium sp. S23321	7.927849979
Burkholderia sp. CCGE1002	7.821025748
Candidatus Amoebophilus asiaticus	7.812664685
Echinicola vietnamensis	7.774141274
Streptomyces cattleya	7.738062424
Gloeobacter violaceus	7.731351933
Saprospira grandis	7.692614376
Ignavibacterium album	7.629801766
Methylotenera versatilis	7.601289989
Blattabacterium sp. (Nauphoeta cinerea)	7.557020871
Pseudomonas resinovorans	7.495473892
Thermotoga thermarum	7.488549256
Spirochaeta sp. L21-RPul-D2	7.327316828
Candidatus Liberibacter asiaticus	7.3199639
Corynebacterium ulcerans	7.314047399

Methylibium petroleiphilum	7.125482124
Staphylococcus pseudintermedius	7.091438787
Hyposoter fugitivus ichnovirus	7.055359937
Nostoc sp. PCC 7120	7.041296519
Syntrophus aciditrophicus	6.947313425
Anabaena sp. 90	6.944055631
Helicobacter hepaticus	6.851508964
Amycolatopsis orientalis	6.812771406
Natranaerobius thermophilus	6.785866851
Saccharophagus degradans	6.784430425
Lactobacillus buchneri	6.77323986
Desulfovibrio africanus	6.752831649
Thioalkalivibrio nitratireducens	6.742649219
Solenopsis invicta virus 3	6.677007107
Xanthobacter autotrophicus	6.669868325
Yersinia pestis	6.664165969
Methanosarcina barkeri	6.637047269
Streptomyces hygroscopicus	6.611364994
Hyphomonas neptunium	6.558778165
Methylobacillus flagellatus	6.500274834
Cupriavidus necator	6.455620775
Emticicia oligotrophica	6.420355158
Chlamydia psittaci	6.414438657
Marinobacter sp. BSs20148	6.397117445
Bdellovibrio bacteriovorus	6.337391833
Desulfotomaculum carboxydivorans	6.310487278
Actinosynnema mirum	6.277452076
Parvularcula bermudensis	6.227309808
Cytophaga hutchinsonii	6.120485577
Spiribacter salinus	6.097247864
Cupriavidus taiwanensis	6.083184446
Streptococcus intermedius	6.083184446
Pantoea vagans	6.038744533
Mycoplasma gallisepticum	6.030597616
Burkholderia sp. YI23	6.024466969
Aquifex aeolicus	5.979018922
Shewanella pealeana	5.926217946
Paramecium bursaria Chlorella virus 1	5.908682589
Candidatus Desulforudis audaxviator	5.904801601
Blastococcus saxobsidens	5.847734697
Shewanella halifaxensis	5.833457133
Aureococcus anophagefferens virus	5.830199339
Mycoplasma bovis	5.737866817
Shewanella oneidensis	5.706053896
Candidatus Nasuia deltocephalinicola	5.686073977
Actinoplanes sp. N902-109	5.660391702
Rhodococcus hoagii	5.627570646
Sulfurovum sp. NBC37-1	5.572110964
Phycisphaera mikurensis	5.555003897
Agrobacterium vitis	5.490798211
Acidithiobacillus caldus	5.477742929
Zunongwangia profunda	5.476520648
Desulfurobacterium thermolithotrophum	5.470604147

Roseburia intestinalis	5.470604147
Azospira oryzae	5.45898529
Brevundimonas subvibrioides	5.451846508
Gemmatimonas aurantiaca	5.43778309
Arcobacter nitrofigilis	5.432294881
Shewanella woodyi	5.426164234
Zobellia galactanivorans	5.41066439
Bradyrhizobium oligotrophicum	5.406184315
Rhodobacter capsulatus	5.399259679
Bifidobacterium animalis	5.373363259
Shewanella amazonensis	5.354819766
Gordonibacter pamelaeae	5.345022277
Streptococcus phage Cp-1	5.317903576
Erwinia amylovora	5.185012129
Granulicella tundricola	5.137913509
Thermosynechococcus elongatus	5.1324253
Sphingomonas sp. MM-1	5.126294653
Sulfurospirillum deleyianum	5.10652888
Lactobacillus plantarum	5.066569042
Acinetobacter sp. ADP1	5.003371491
Salinispora tropica	4.989308073
Actinoplanes sp. SE50/110	4.970550434
Arcobacter sp. L	4.969328154
Acidovorax sp. KKS102	4.949348235
Turneriella parva	4.923880106
Shewanella frigidimarina	4.897983686
Polaromonas naphthalenivorans	4.889408477
Pelotomaculum thermopropionicum	4.826639218
Calyptogena okutanii thioautotrophic gill symbiont	4.820722717
Streptomyces coelicolor	4.806445153
Carboxydothermus hydrogenoformans	4.760997105
Paenibacillus larvae	4.73958076
Singulisphaera acidiphila	4.643776298
Methylomonas methanica	4.638073943
Conexibacter woesei	4.630935161
Simkania negevensis	4.596677678
Streptomyces scabiei	4.583836541
Candidatus Moranella endobia	4.578134186
Salinispora arenicola	4.558154267
Blattabacterium punctulatus	4.552451911
Oscillatoria acuminata	4.551015485
Fluviicola taffensis	4.545313129
Wolbachia endosymbiont of Onchocerca ochengi	4.512492073
Raoultella ornithinolytica	4.508011999
Sulfurimonas denitrificans	4.506789718
Rivularia sp. PCC 7116	4.505353291
Anaplasma marginale	4.442369886
Glaciecola sp. 4H-3-7+YE-5	4.377950054
Sorangium cellulosum	4.356747854
Dyadobacter fermentans	4.345128998
Alteromonas sp. SN2	4.325363225
Rhizobium sp. IRBG74	4.312307942
Burkholderia sp. RPE64	4.253804611

Corallococcus coralloides	4.240963474
Robiginitalea biformata	4.20548371
Bacteroides xylanisolvens	4.186940218
Runella slithyformis	4.179801436
Azoarcus sp. KH32C	4.168182579
Desulfobacula toluolica	4.154119162
Bacillus clausii	4.141278024
Brachyspira pilosicoli	4.11559575
Anaeromyxobacter dehalogenans	4.108456968
Enterobacter lignolyticus	4.096838112
Aromatoleum aromaticum	4.089913475
Corynebacterium callunae	4.031195999
Choristoneura occidentalis granulovirus	4.019577143
Croceibacter atlanticus	3.978395024
Candidatus Babela massiliensis	3.965553887
Erythrobacter litoralis	3.95393503
Oligotropha carboxidovorans	3.95393503
Azospirillum lipoferum	3.941093893
Desulfitobacterium hafniense	3.927030475
Blattabacterium sp. (Panesthia angustipennis spadica)	3.905614129
Candidatus Pelagibacter sp. IMCC9063	3.888292918
Streptomyces davawensis	3.882590562
gamma proteobacterium HdN1	3.81694845
Flavobacterium johnsoniae	3.815726169
Desulfobacca acetoxidans	3.809809668
Neorickettsia sennetsu	3.802885032
Alistipes finegoldii	3.790043895
Dehalococcoides mccartyi	3.757008693
Nitrosospira multiformis	3.751306337
Phenylobacterium zucineum	3.744167555
Hydrogenobaculum sp. Y04AAS1	3.724187636
Halothece sp. PCC 7418	3.718485281
Streptomyces collinus	3.704207717
Halomonas elongata	3.674045368
Desulfovibrio alaskensis	3.645704387
Burkholderia xenovorans	3.641224312
Maruca vitrata nucleopolyhedrovirus	3.628383175
Bradyrhizobium sp. ORS 278	3.615542038
Halovivax ruber	3.615542038
Zymomonas mobilis	3.615542038
Gloeobacter kilaueensis	3.60147862
Methanococcus voltae	3.595562118
Methylacidiphilum infernorum	3.594339838
Methylobacterium nodulans	3.582720981
Allochromatium vinosum	3.575582199
Polynucleobacter necessarius	3.569879844
Acidovorax ebreus	3.562741062
Polaromonas sp. JS666	3.562741062
Pandoraea sp. RB-44	3.549899925
Nostoc sp. PCC 7524	3.4398179
Simiduia agarivorans	3.4398179
Geobacillus thermodenitrificans	3.425754482
Azotobacter vinelandii	3.406996844

Listeria innocua	3.392933426
Nostoc sp. PCC 7107	3.391496999
Nitrosococcus halophilus	3.38723107
Mycobacterium gilvum	3.36133465
Bacillus anthracis	3.321588958
Xenorhabdus nematophila	3.30874782
Burkholderia sp. CCGE1003	3.308533675
Thermus thermophilus	3.295906683
Agrobacterium fabrum	3.2828514
Akkermansia muciniphila	3.270010263
Xanthomonas albilineans	3.270010263
Cyanothece sp. PCC 7424	3.257169126
Phaeocystis globosa virus	3.237189207
Methanothermococcus okinawensis	3.217209288
Nocardia cyriacigeorgica	3.211506932
Rhodomicrobium vannielii	3.211506932
Erwinia tasmaniensis	3.178685876
Pectobacterium atrosepticum	3.164408312
Calothrix sp. PCC 7507	3.118746119
Pseudomonas sp. UW4	3.081444988
Lactobacillus helveticus	3.080222707
Pseudomonas entomophila	3.074520352
Vicia cryptic virus	3.061465069
Methylocystis sp. SC2	3.054540433
Streptomyces violaceusniger	3.048623932
Nitratifractor salsuginis	3.041699296
Leuconostoc gasicomitatum	3.040262869
Morganella morganii	3.035782795
Salinarchaeum sp. Harcht-Bsk1	3.035782795
Human herpesvirus 7	3.015802876
Desulfosporosinus orientis	2.995822957
Candidatus Arthromitus sp. SFB-mouse	2.976057183
Choristoneura rosaceana entomopoxvirus 'L'	2.924478489
Orientia tsutsugamushi	2.911637351
Mycobacterium bovis	2.90449857
Desulfovibrio desulfuricans	2.878816295
Thioflavicoccus mobilis	2.878816295
Bartonella australis	2.845995239
Rhizobium leguminosarum	2.833154102
Desulfosporosinus meridiei	2.82601532
Papaya leaf curl alphasatellite	2.814396463
Blattabacterium sp. (Blaberus giganteus)	2.813174183
Bacillus licheniformis	2.798896619
Mycobacterium chubuense	2.781575407
Pseudovibrio sp. FO-BEG1	2.76873427
Vibrio tasmaniensis	2.76873427
Thermobaculum terrenum	2.767511989
Chloroherpeton thalassium	2.761809634
Desulfococcus oleovorans	2.761809634
Desulfovibrio aespoeensis	2.761809634
Pseudoxanthomonas suwonensis	2.748754351
Dactylococcopsis salina	2.74753207
Pseudomonas brassicacearum	2.735913214

Vibrio nigripulchritudo	2.735913214
Rhodospirillum centenum	2.723072077
Mycoplasma synoviae	2.696167521
Staphylothermus hellenicus	2.683326384
Cyprinid herpesvirus 2	2.677409883
Ehrlichia ruminantium	2.677409883
Deinococcus radiodurans	2.670271101
Bordetella pertussis	2.637450045
Synechococcus sp. JA-2-3B'a(2-13)	2.624608908
Nautilia profundicola	2.611767771
Thalassolituus oleivorans	2.591787851
Asticcacaulis excentricus	2.586085496
Leptospira biflexa	2.571807932
Desulfotomaculum kuznetsovii	2.566105577
Rhodococcus jostii	2.538986876
Streptococcus phage SM1	2.513304602
Nitrobacter winogradskyi	2.501685745
Pseudomonas protegens	2.501685745
Streptococcus phage PH10	2.480483545
Xanthomonas oryzae	2.468864689
Caldisericum exile	2.467642408
Shewanella denitrificans	2.467642408
Stanieria cyanosphaera	2.456023552
Chlorobium limicola	2.423202496
Acidovorax avenae	2.410361358
Sphingomonas wittichii	2.410361358
Parvibaculum lavamentivorans	2.390381439
Planctomyces brasiliensis	2.390381439
Campylobacter lari	2.344719246
Candidatus Nitrospira defluvii	2.324739327
Meiothermus ruber	2.31189819
Haliscomenobacter hydrossis	2.266235996
Nonlabens dokdonensis	2.220573803
Thermotoga lettingae	2.213435021
Thermacetogenium phaeum	2.207732665
Sulfuricurvum kujiense	2.200593884
Cercopithecine herpesvirus 2	2.188975027
Laodelphax striatella honeydew virus 1	2.188975027
Prunus necrotic ringspot virus	2.188975027
Spiribacter sp. UAH-SP71	2.188975027
Granulicella mallensis	2.156153971
Terriglobus roseus	2.149229335
Burkholderia sp. CCGE1001	2.143312834
Cynomolgus macaque cytomegalovirus strain Ottawa	2.143312834
Methylobacterium sp. 4-46	2.143312834
Fervidobacterium pennivorans	2.123332915
Arthrobacter aurescens	2.09765064
Hyphomicrobium sp. MC1	2.09765064
Burkholderia vietnamiensis	2.090511858
Yersinia phage phiA1122	2.064829584
Beijerinckia indica	2.051988447
Lactobacillus rhamnosus	2.051988447
Actinoplanes missouriensis	2.044849665

Bacillus halodurans	2.044849665
candidate division WWE3 bacterium RAAC2_WWE3_1	2.032008528
Mycobacterium ulcerans	2.01916739
Deinococcus maricopensis	2.006326253
Helicobacter felis	1.96066406
Rhodobacter sphaeroides	1.959227633
Micavibrio aeruginosavorus	1.953525278
Haemophilus phage Aaphi23	1.88218081
Actinomyces phage Av-1	1.876264309
Cupriavidus pinatubonensis	1.876264309
Melon yellow spot virus	1.876264309
Novosphingobium sp. PP1Y	1.876264309
Thermus scotoductus	1.876264309
Helicobacter mustelae	1.869339673
Sphingobium sp. SYK-6	1.843443253
Enterobacteriaceae bacterium strain FGI 57	1.830602116
Methanosarcina acetivorans	1.830602116
Pseudomonas fulva	1.830602116
Candidatus Accumulibacter phosphatis	1.810622196
Corynebacterium terpenotabidum	1.810622196
Chloroflexus aggregans	1.797781059
Borrelia crocidurae	1.784939922
Cyanobium gracile	1.784939922
Mycoplasma haemofelis	1.784939922
Modestobacter marinus	1.77780114
Candidatus Kinetoplastibacterium crithidii	1.739277729
Deinococcus geothermalis	1.71929781
Chlamydia pneumoniae	1.712159028
Nocardia brasiliensis	1.69931789
Geobacter sulfurreducens	1.693615535
Maricaulis maris	1.693615535
Nitrosomonas europaea	1.693615535
Rhodococcus opacus	1.693615535
Clostridium ljungdahlii	1.686476753
Pseudomonas syringae	1.686476753
Exiguobacterium sibiricum	1.647953342
Nitrobacter hamburgensis	1.563553591
Pseudoxanthomonas spadix	1.563553591
Psychrobacter arcticus	1.563553591
Sinorhizobium fredii	1.563553591
Thermoplasma volcanium	1.563553591
Acidovorax sp. JS42	1.530732535
Anaeromyxobacter sp. Fw109-5	1.530732535
Candidatus Kinetoplastibacterium desouzaii	1.530732535
Cronobacter sakazakii	1.530732535
Exiguobacterium sp. MH3	1.530732535
Trichodesmium erythraeum	1.517891397
Ictalurid herpesvirus 1	1.497911478
Methylotenera mobilis	1.485070341
Spiroplasma taiwanense	1.485070341
Aeromonas veronii	1.472229204
Anabaena variabilis	1.472229204
Hahella chejuensis	1.472229204

Pseudomonas poae	1.472229204
Borrelia recurrentis	1.465090422
Streptomyces albus	1.465090422
Verrucosispora maris	1.465090422
Yersinia pseudotuberculosis	1.465090422
Mycobacterium vanbaalenii	1.452249285
Thiomonas intermedia	1.452249285
Mycoplasma mycoides	1.439408148
Wolinella succinogenes	1.439408148
Bartonella quintana	1.419428229
Burkholderia phenoliruptrix	1.406587091
Mesorhizobium ciceri	1.393745954
Geobacillus sp. WCH70	1.386607172
Dickeya zeae	1.380904817
Blattabacterium sp. (Periplaneta americana)	1.373766035
Mycoplasma agalactiae	1.373766035
Thermodesulfovibrio yellowstonii	1.360924898
Candidatus Arthromitus sp. SFB-rat-Yit	1.348083761
Candidatus Phytoplasma solani	1.348083761
Sulfurimonas autotrophica	1.348083761
Aggregatibacter phage S1249	1.335242623
Brachyspira intermedia	1.335242623
Synechococcus sp. CC9605	1.335242623
Caulobacter sp. K31	1.250842873
Chlorobium phaeovibrioides	1.250842873
Emiliania huxleyi virus 86	1.250842873
Enterobacter aerogenes	1.250842873
Frankia sp. CcI3	1.250842873
Frankia sp. EAN1pec	1.250842873
Human herpesvirus 6B	1.250842873
Shewanella sp. MR-7	1.250842873
Sphingobium japonicum	1.250842873
Candidatus Chloracidobacterium thermophilum	1.218021816
Eel picornavirus 1	1.218021816
Aeromonas hydrophila	1.205180679
Rhizoctonia solani dsRNA virus 2	1.205180679
Thermomicrobium roseum	1.205180679
Desulfovibrio magneticus	1.172359623
Enterobacter sp. R4-368	1.172359623
Sphaerochaeta pleomorpha	1.172359623
Blattabacterium sp. (Blattella germanica)	1.159518486
Methylocella silvestris	1.159518486
Pseudomonas monteilii	1.159518486
Chelativorans sp. BNC1	1.152379704
beta proteobacterium CB	1.139538567
Desulfocapsa sulfexigens	1.139538567
Arthrobacter sp. Rue61a	1.126697429
Pelobacter carbinolicus	1.126697429
Pseudoalteromonas sp. SM9913	1.126697429
Methanohalophilus mahii	1.119558648
Mycoplasma conjunctivae	1.119558648
Saccharothrix espanaensis	1.119558648
Streptococcus phage EJ-1	1.119558648

Calothrix parietina	1.113856292
Stigmatella aurantiaca	1.113856292
Thermosynechococcus sp. NK55a	1.113856292
Aeromonas phage phiAS5	1.10671751
Clostridium phage c-st	1.10671751
Methylovorus glucosotrophus	1.093876373
Ruegeria sp. TM1040	1.093876373
Wolbachia endosymbiont of Drosophila simulans	1.093876373
Lacinutrix sp. 5H-3-7-4	1.081035236
Octadecabacter antarcticus	1.081035236
Sulfurospirillum barnesii	1.081035236
Aeromonas salmonicida	1.068194099
Deinococcus peraridilitoris	1.068194099
Acidovorax citrulli	0.938132154
Aquamavirus A	0.938132154
Bordetella bronchiseptica	0.938132154
Candidatus Nitrososphaera gargensis	0.938132154
Citrobacter rodentium	0.938132154
Cryptophlebia leucotreta granulovirus	0.938132154
Erinnyis ello granulovirus	0.938132154
Halorubrum lacusprofundi	0.938132154
Marinomonas mediterranea	0.938132154
Methanothermus fervidus	0.938132154
Paracoccus aminophilus	0.938132154
Polymorphum gilvum	0.938132154
Rose rosette virus	0.938132154
Rosellinia necatrix partitivirus 2	0.938132154
Salmonella bongori	0.938132154
Streptococcus phage Dp-1	0.938132154
Alcanivorax dieselolei	0.905311098
Frankia alni	0.905311098
Pseudomonas sp. TKP	0.905311098
Pseudomonas sp. VLB120	0.905311098
Chamaesiphon minutus	0.892469961
Geobacter sp. M18	0.892469961
Methylovorus sp. MP688	0.892469961
Stenotrophomonas phage S1	0.892469961
Streptomyces fulvissimus	0.892469961
Anabaena cylindrica	0.872490042
Enterobacter asburiae	0.872490042
Neorickettsia risticii	0.872490042
Streptomyces sp. SirexAA-E	0.872490042
Caulobacter vibrioides	0.859648905
Ectocarpus siliculosus virus 1	0.859648905
Geobacillus sp. C56-T3	0.846807768
Mycobacterium avium	0.846807768
Shewanella violacea	0.846807768
Thermoanaerobacter italicus	0.846807768
Brachyspira hyodysenteriae	0.839668986
Erwinia billingiae	0.839668986
Desulfitobacterium dehalogenans	0.813986711
Helicobacter acinonychis	0.813986711
Acaryochloris marina	0.801145574

Geodermatophilus obscurus	0.801145574
Pelagibacterium halotolerans	0.801145574
Prosthecochloris aestuarii	0.801145574
Rickettsia akari	0.801145574
Spiroplasma apis	0.801145574
Sulfurihydrogenibium sp. YO3AOP1	0.801145574
Acidithiobacillus ferrooxidans	0.625421436
Alcanivorax borkumensis	0.625421436
Azorhizobium caulinodans	0.625421436
Candidatus Koribacter versatilis	0.625421436
Deinococcus deserti	0.625421436
Edwardsiella ictaluri	0.625421436
Glypta fumiferanae ichnovirus	0.625421436
Grapevine Syrah virus 1	0.625421436
Haloquadratum walsbyi	0.625421436
Marinobacter adhaerens	0.625421436
Methanobrevibacter ruminantium	0.625421436
Methanococcus vannielii	0.625421436
Methanomethylovorans hollandica	0.625421436
Myxococcus xanthus	0.625421436
Rhizobium tropici	0.625421436
Saimiriine herpesvirus 1	0.625421436
Stenotrophomonas phage phiSMA7	0.625421436
Streptococcus phage IC1	0.625421436
Streptococcus phage TP-778L	0.625421436
Alcelaphine herpesvirus 1	0.59260038
Bdellovibrio exovorus	0.59260038
Candidatus Kinetoplastibacterium oncopeltii	0.59260038
Hyphomicrobium denitrificans	0.59260038
Rhizobium etli	0.59260038
Chlamydia pecorum	0.579759243
Desulfohalobium retbaense	0.579759243
Erwinia sp. Ejp617	0.579759243
Geobacter sp. M21	0.579759243
Gloeocapsa sp. PCC 7428	0.579759243
Leptospira interrogans	0.579759243
Onion yellows phytoplasma	0.579759243
Streptococcus phage DCC1738	0.579759243
Tomato zonate spot virus	0.579759243
Waddlia chondrophila	0.579759243
Xanthomonas alfalfae	0.579759243
Yaba monkey tumor virus	0.579759243
Arthrospira platensis	0.559779324
Bartonella bacilliformis	0.559779324
Ehrlichia muris	0.559779324
Fowl aviadenovirus D	0.559779324
Macacine herpesvirus 1	0.559779324
Mesorhizobium opportunistum	0.559779324
Pelodictyon luteolum	0.559779324
Pelodictyon phaeoclathratiforme	0.559779324
Psychrobacter cryohalolentis	0.559779324
Rhodopirellula baltica	0.559779324
Synechococcus sp. JA-3-3Ab	0.559779324

Tistrella mobilis	0.559779324
Bacillus sp. JS	0.546938187
Candidatus Methanomassiliicoccus intestinalis	0.546938187
Desulfovibrio gigas	0.546938187
Microcoleus sp. PCC 7113	0.546938187
Serratia liquefaciens	0.546938187
Shewanella putrefaciens	0.546938187
Sphaerochaeta coccoides	0.546938187
Bartonella tribocorum	0.534097049
Caldicellulosiruptor kristjanssonii	0.534097049
Chlamydia muridarum	0.534097049
Listeria seeligeri	0.534097049
Mycoplasma crocodyli	0.534097049
Paracoccus denitrificans	0.534097049
Streptomyces rapamycinicus	0.534097049
Ureaplasma parvum	0.534097049
Agrobacterium sp. H13-3	0.312710718
Agrobacterium tumefaciens	0.312710718
Agrotis ipsilon multiple nucleopolyhedrovirus	0.312710718
Bartonella vinsonii	0.312710718
Bovine herpesvirus 5	0.312710718
Candidatus Atelocyanobacterium thalassa	0.312710718
Candidatus Blochmannia vafer	0.312710718
Candidatus Liberibacter americanus	0.312710718
Caulobacter segnis	0.312710718
Chrysodeixis chalcites nucleopolyhedrovirus	0.312710718
Cotesia congregata bracovirus	0.312710718
Desulfovibrio hydrothermalis	0.312710718
Dinoroseobacter shibae	0.312710718
Enterococcus sp. 7L76	0.312710718
Francisella noatunensis	0.312710718
Francisella philomiragia	0.312710718
Goatpox virus	0.312710718
Halomicrobium mukohataei	0.312710718
Human herpesvirus 5	0.312710718
Human mastadenovirus C	0.312710718
Invertebrate iridescent virus 31	0.312710718
Jannaschia sp. CCS1	0.312710718
Leptolyngbya sp. PCC 7376	0.312710718
Leuconostoc sp. C2	0.312710718
Mesorhizobium australicum	0.312710718
Methanobrevibacter smithii	0.312710718
Methanocella conradii	0.312710718
Methanosaeta harundinacea	0.312710718
Natronococcus occultus	0.312710718
Nitrosococcus watsonii	0.312710718
Ovine herpesvirus 2	0.312710718
Penaeus monodon nudivirus	0.312710718
Phaseolus vulgaris endornavirus	0.312710718
Rice grassy stunt virus	0.312710718
Saimiriine herpesvirus 4	0.312710718
Sinorhizobium medicae	0.312710718
Staphylococcus pasteuri	0.312710718

Streptococcus phage YMC-2011	0.312710718
Streptomyces sp. PAMC26508	0.312710718
Synechocystis sp. PCC 6803	0.312710718
Thermaerobacter marianensis	0.312710718
Thermosipho africanus	0.312710718
Thermotoga maritima	0.312710718
Thottapalayam virus	0.312710718
Vernonia yellow vein Fujian virus betasatellite	0.312710718
White spot syndrome virus	0.312710718
Xanthomonas euvesicatoria	0.312710718
Acanthocystis turfacea Chlorella virus 1	0.279889662
Acidiphilium cryptum	0.279889662
Acidobacterium capsulatum	0.279889662
Archaeoglobus fulgidus	0.279889662
Bacillus atrophaeus	0.279889662
Borrelia miyamotoi	0.279889662
Candidatus Blochmannia chromaiodes	0.279889662
Candidatus Midichloria mitochondrii	0.279889662
Cercopithecine herpesvirus 5	0.279889662
Chimpanzee alpha-1 herpesvirus	0.279889662
Chlorobium phaeobacteroides	0.279889662
Clostridium autoethanogenum	0.279889662
Coraliomargarita akajimensis	0.279889662
Escherichia fergusonii	0.279889662
Exiguobacterium antarcticum	0.279889662
Invertebrate iridescent virus 6	0.279889662
Methanocaldococcus infernus	0.279889662
Methanococcoides burtonii	0.279889662
Methanoculleus marisnigri	0.279889662
Mycobacterium intracellulare	0.279889662
Mycoplasma capricolum	0.279889662
Pseudomonas phage phi297	0.279889662
Rickettsia rhipicephali	0.279889662
Shewanella sp. W3-18-1	0.279889662
Thermosipho melanesiensis	0.279889662
Trichormus azollae	0.279889662
Wheat streak mosaic virus	0.279889662
Acidilobus saccharovorans	0.267048525
Aciduliprofundum sp. MAR08-339	0.267048525
Desulfatibacillum alkenivorans	0.267048525
Ehrlichia chaffeensis	0.267048525
Geobacillus thermoleovorans	0.267048525
Gordonia bronchialis	0.267048525
Halogeometricum borinquense	0.267048525
Lactobacillus casei	0.267048525
Lactobacillus paracasei	0.267048525
Liberibacter crescens	0.267048525
Methanosaeta thermophila	0.267048525
Mycobacterium marinum	0.267048525
Pelobacter propionicus	0.267048525
Roseiflexus castenholzii	0.267048525
Shewanella sediminis	0.267048525
Streptococcus phage K13	0.267048525

Suid herpesvirus 1	0.267048525
Sulfurihydrogenibium azorense	0.267048525
Synechococcus sp. PCC 6312	0.267048525
Synechococcus sp. WH 7803	0.267048525
Synechococcus sp. WH 8102	0.267048525
Vibrio alginolyticus	0.267048525
Vibrio sp. Ex25	0.267048525

NAseq in OLP patients and healthy individuals	
Healthy Control	
**Microbiome Species**	**Average Counts (normalized)**
Veillonella parvula	3724871.081
Rothia mucilaginosa	3400094.702
Neisseria meningitidis	588859.8523
Achromobacter xylosoxidans	437267.0777
Fusobacterium nucleatum	353136.9482
Haemophilus parainfluenzae	316376.3645
Campylobacter concisus	117352.6669
Prevotella melaninogenica	97702.18592
Streptococcus parasanguinis	86517.21503
Selenomonas sputigena	45974.20639
Haemophilus influenzae	44555.85145
Rothia dentocariosa	42870.05608
Atopobium parvulum	39603.98125
Leptotrichia buccalis	35762.97248
Megasphaera elsdenii	28452.14891
Streptococcus mitis	26744.71887
Hyphomicrobium nitrativorans	25381.94056
Streptococcus pneumoniae	22132.21855
Aggregatibacter aphrophilus	21874.52533
Selenomonas ruminantium	21136.28642
Streptococcus pyogenes	21028.21579
Streptococcus oligofermentans	20861.04881
Pseudogulbenkiania sp. NH8B	19012.63351
Porphyromonas asaccharolytica	17612.37212
Streptococcus constellatus	16750.70875
Tannerella forsythia	16560.70017
Dichelobacter nodosus	14403.63067
Taylorella equigenitalis	12259.22948
Streptococcus dysgalactiae	12199.90474
Prevotella intermedia	11708.79333
Capnocytophaga ochracea	11180.09989
Neisseria lactamica	9853.277387
Candidatus Saccharimonas aalborgensis	9621.6254
[Eubacterium] eligens	9541.268338
Mannheimia haemolytica	9187.991104
Streptococcus sanguinis	8994.986067
Prevotella sp. oral taxon 299	8615.378326
Riemerella anatipestifer	8418.870748
[Clostridium] saccharolyticum	7866.75479
Kocuria rhizophila	7733.736486
Magnetococcus marinus	7368.207455
Alteromonas macleodii	7234.179811
Campylobacter jejuni	7018.583919
Eubacterium rectale	6690.587909
Clostridium sp. SY8519	6251.304172
Propionibacterium avidum	6030.84647
Jonesia denitrificans	5765.872779
Mobiluncus curtisii	5570.453145
Leadbetterella byssophila	5295.564957
Escherichia coli	5173.880001

Streptococcus pseudopneumoniae	4980.888944
Streptococcus equi	4830.356386
Bacteroides vulgatus	4717.370455
Beutenbergia cavernae	4495.038065
Streptococcus sp. I-P16	4389.27256
Lachnoclostridium phytofermentans	4316.653098
butyrate-producing bacterium SSC/2	4312.543628
Flavobacteriaceae bacterium 3519-10	4113.611124
Macrococcus caseolyticus	4076.907326
Thauera sp. MZ1T	4027.346287
Chitinophaga pinensis	4009.422643
Corynebacterium argentoratense	4003.707466
Mycoplasma hyopneumoniae	3782.295088
Paludibacter propionicigenes	3781.083388
Coprococcus catus	3689.858213
Niastella koreensis	3160.068531
Neisseria gonorrhoeae	3082.300459
Porphyromonas gingivalis	3082.158003
Brachybacterium faecium	3070.00558
Streptococcus gordonii	2757.068213
Marivirga tractuosa	2536.469026
Campylobacter curvus	2412.02812
Pedobacter heparinus	2213.08841
Carnobacterium maltaromaticum	2123.228035
Candidatus Saccharibacteria bacterium RAAC3_TM7_1	2102.107879
Thiocystis violascens	2057.371828
Ruminiclostridium thermocellum	2048.161031
Staphylococcus haemolyticus	2007.143841
Clostridium acetobutylicum	1996.934232
Kribbella flavida	1900.031785
Prevotella ruminicola	1893.32894
Campylobacter hominis	1813.711047
Lactobacillus fermentum	1785.793544
[Clostridium] stercorarium	1763.357169
Halanaerobium hydrogeniformans	1738.783522
Roseburia hominis	1727.431342
[Eubacterium] siraeum	1696.004277
Syntrophobacter fumaroxidans	1665.604872
Streptococcus sp. I-G2	1610.082493
Alkaliphilus metalliredigens	1592.506089
Streptococcus salivarius	1590.901435
Azoarcus sp. BH72	1560.033169
Methylophaga nitratireducenticrescens	1508.359836
Aggregatibacter actinomycetemcomitans	1505.691993
Arcanobacterium haemolyticum	1485.478593
Bibersteinia trehalosi	1450.248625
Campylobacter fetus	1419.002542
Streptococcus macedonicus	1397.518183
Tick-borne encephalitis virus	1378.887338
Butyrivibrio proteoclasticus	1374.303182
Ralstonia solanacearum	1326.811592
Collimonas fungivorans	1241.451202
Sebaldella termitidis	1231.364165

Streptococcus suis	1209.310297
Jingmen tick virus	1203.381553
Bronchotrix virus Thermophacta bacteriphage	1199.944349
Thiobacillus denitrificans	1197.759013
Thioalkalivibrio sulfidiphilus	1195.614474
Bacillus virus SPO1	1183.753657
Clostridium botulinum	1175.968151
Peptoclostridium difficile	1119.425713
Comamonas testosteroni	1091.797667
Acidothermus cellulolyticus	1090.659401
Pseudonocardia dioxanivorans	1089.683468
Saccharopolyspora erythraea	1063.587127
Mycobacterium sp. JDM601	1013.647973
Ilyobacter polytropus	962.0787043
Lactobacillus reuteri	938.6877164
Capnocytophaga canimorsus	924.0536112
Streptococcus uberis	910.1287803
Pasteurella multocida	900.8000043
Anaplasma centrale	882.1163733
Actinobacillus succinogenes	872.7187188
Streptococcus anginosus	869.5390025
[Mannheimia] succiniciproducens	864.5040963
Tetragenococcus halophilus	856.9720045
Geitlerinema sp. PCC 7407	843.7771703
[Ruminococcus] torques	840.8249326
Micrococcus luteus	823.4066623
Oscillibacter valericigenes	812.563426
Parabacteroides distasonis	806.1804601
Megamonas hypermegale	795.9129307
Leptothrix cholodnii	772.9800965
Rhodospirillum rubrum	739.2925072
Bacillus cytotoxicus	738.9923677
Candidatus Carsonella ruddii	660.3265729
Candidatus Puniceispirillum marinum	652.3874627
Prevotella dentalis	622.9592803
Corynebacterium diphtheriae	612.8530431
Mycoplasma hyorhinis	595.5852883
Nocardiopsis alba	594.9108397
Bacillus thuringiensis	590.5780325
Frateuria aurantia	590.5309074
Enterococcus hirae	576.4713361
Haemophilus parasuis	571.6545051
Solitalea canadensis	562.9073323
Gramella forsetii	537.6464659
Oceanithermus profundus	535.2875811
Clostridium tetani	520.5732011
Streptococcus agalactiae	494.4126379
Staphylococcus epidermidis	484.0694306
Sodalis glossinidius	479.2489577
Sulfurimonas denitrificans	477.5548574
Olsenella uli	477.3555713
Corynebacterium efficiens	473.1340779
Nocardia farcinica	472.9352812

Pseudomonas aeruginosa	472.0516469
Methylomicrobium alcaliphilum	442.0175434
Planctomyces limnophilus	441.458204
Caldicellulosiruptor kronotskyensis	436.1607413
Flavobacterium indicum	422.3165745
Actinobacillus pleuropneumoniae	412.886012
Segniliparus rotundus	396.9615908
Thioalkalimicrobium cyclicum	393.9781366
Histophilus somni	385.1014007
Clostridium kluyveri	347.1385217
Dill cryptic virus 2	338.6464254
Actinobacillus suis	329.5166589
Dickeya dadantii	327.4899538
Streptococcus thermophilus	313.2142669
Treponema denticola	312.2699798
Candidatus Zinderia insecticola	308.4677183
Spring beauty latent virus	302.6421953
Streptococcus mutans	295.5675
Haemophilus ducreyi	294.4475409
Lactococcus garvieae	292.4641919
Desulfurispirillum indicum	280.6237649
Mahella australiensis	275.6328761
Alkalilimnicola ehrlichii	265.1045754
Lactobacillus sanfranciscensis	263.3641413
Propionibacterium propionicum	255.7635965
Amycolicicoccus subflavus	254.2935929
Buchnera aphidicola	254.0760216
Candidatus Azobacteroides pseudotrichonymphae	250.5065662
Kinetoplastibacterium blastocrithidii	248.6164897
Treponema pedis	244.818501
Cellulosilyticum lentocellum	243.002531
Cryptobacterium curtum	241.8045812
Sulfobacillus acidophilus	240.9539418
Flavobacterium branchiophilum	233.7224899
Bacillus subtilis	232.8633112
Mouse astrovirus M-52/USA/2008	229.1894991
Tsukamurella paurometabola	206.0722012
Helicobacter cinaedi	200.3456722
Owenweeksia hongkongensis	199.6222087
Amphibacillus xylanus	198.5334131
Aerococcus urinae	198.0931861
Methanococcus maripaludis	193.4058905
Ethanoligenens harbinense	192.3229931
Halothiobacillus neapolitanus	189.2970059
Eubacterium limosum	177.5542408
Coprococcus sp. ART55/1	172.7705604
Anaerococcus prevotii	160.7524485
Burkholderia multivorans	157.900127
Stenotrophomonas maltophilia	151.3554265
Bifidobacterium thermophilum	149.1115916
Desulfomonile tiedjei	148.1586541
Candidatus Methylomirabilis oxyfera	145.9763262
Gallibacterium anatis	145.8546676

Lactobacillus salivarius	140.6022544
Frankia sp. EuI1c	137.96555
Filifactor alocis	134.7828868
Halobacteroides halobius	133.1329501
Streptobacillus moniliformis	131.3285733
Erysipelothrix rhusiopathiae	125.9551407
Candidatus Sulcia muelleri	124.2681865
Bacillus coagulans	121.3731777
Salinibacter ruber	119.0097034
Kineococcus radiotolerans	117.7624774
Sphingobacterium sp. 21	117.0316585
Lactococcus lactis	116.5067402
Clostridium perfringens	111.2642735
Burkholderia gladioli	107.2308349
Flexistipes sinusarabici	103.8389915
Candidatus Baumannia cicadellinicola	103.6381554
Meiothermus silvanus	103.4068339
Pedobacter saltans	103.2453682
[Clostridium] sticklandii	102.1414601
Mycobacterium kansasii	101.2542022
Propionibacterium acnes	100.5032214
Desulfitobacterium dichloroeliminans	100.4663668
Streptococcus iniae	99.80942201
Streptococcus lutetiensis	99.61262661
Variovorax paradoxus	97.09748344
Candidatus Hamiltonella defensa	97.03381751
Chromobacterium violaceum	96.78156288
Hop trefoil cryptic virus 2	96.37412963
Salmonella enterica	94.7750895
Odoribacter splanchnicus	94.74114756
Gottschalkia acidurici	94.47600146
Allochromatium vinosum	94.10207599
Caldanaerobacter subterraneus	93.83311612
Desulfomicrobium baculatum	92.93661868
Caldicellulosiruptor saccharolyticus	92.74325009
Rhodospirillum photometricum	89.59946302
Prochlorococcus marinus	89.36796972
Streptococcus parauberis	88.73475099
Renibacterium salmoninarum	88.62621866
Faecalitalea cylindroides	88.55822253
Shimwellia blattae	84.18082959
Torque teno midi virus 2	83.5300322
Lysinibacillus sphaericus	82.60557293
Bacteroides thetaiotaomicron	81.85287987
Microbacterium testaceum	79.40482173
Desulfobacterium autotrophicum	79.0300052
Staphylococcus aureus	78.56456703
Syntrophomonas wolfei	77.64259361
Rickettsia australis	75.97795573
Vibrio cholerae	75.43460247
Thermosediminibacter oceani	75.11992755
Staphylococcus lugdunensis	74.93736136
Thermobispora bispora	73.57867516

Rubrobacter xylanophilus	73.2763851
Streptococcus pasteurianus	72.3529166
Corynebacterium kroppenstedtii	72.11215047
Hepatitis C virus	71.57745059
Pseudomonas denitrificans	70.02068128
Treponema primitia	69.21674704
Bacillus amyloliquefaciens	68.72869337
Leptospirillum ferriphilum	68.37758053
Streptococcus infantarius	67.76125183
Bifidobacterium adolescentis	65.14778693
Ruminococcus sp. SR1/5	64.57596544
Arthrobacter arilaitensis	64.57217403
Tropheryma whipplei	64.27830253
Cyanobacterium stanieri	61.62112175
Listeria monocytogenes	61.14054578
Propionibacterium freudenreichii	60.51905797
Ilumatobacter coccineus	60.15106281
Alistipes shahii	59.92181052
Streptomyces bingchenggensis	59.16821398
Serratia symbiotica	58.76493904
Bacteroides fragilis	58.50644713
Desulfotalea psychrophila	57.87321607
Cyanothece sp. PCC 7425	57.41164908
alpha proteobacterium HIMB59	55.79034032
Desulfobulbus propionicus	53.98289354
Bacteroides salanitronis	53.25189255
Treponema brennaborense	53.15195607
Corynebacterium urealyticum	53.04834323
Treponema succinifaciens	51.93913118
Cellulophaga lytica	51.85439233
Thermomonospora curvata	51.71274919
Mycobacterium smegmatis	51.03151424
Clostridium cellulovorans	50.77265543
Thermotoga neapolitana	50.46622709
Acetobacterium woodii	50.32158617
Mycoplasma hominis	50.0689048
Cyclobacterium marinum	50.01642571
Flavobacterium columnare	49.6171317
Lactobacillus kefiranofaciens	49.32197238
Rhodanobacter denitrificans	48.65284759
Aliivibrio fischeri	47.46012468
Caldilinea aerophila	47.25860462
Weissella koreensis	46.5881444
Bacillus cereus	46.58632241
Melissococcus plutonius	46.21683216
Geobacillus sp. GHH01	45.48563394
Treponema azotonutricium	45.32390114
Leifsonia xyli	44.90390112
Enterococcus faecium	44.04443779
Yersinia enterocolitica	43.79692546
Acholeplasma palmae	43.48803352
Corynebacterium matruchotii	42.38345392
Vibrio campbellii	42.28246674

Ammonifex degensii	41.80200813
Novosphingobium aromaticivorans	41.77469032
Mycobacterium abscessus	41.74784675
Faecalibacterium prausnitzii	40.91612566
Spirochaeta thermophila	40.90158187
Aminobacterium colombiense	40.61963091
Ochrobactrum anthropi	40.16552158
Carnobacterium sp. 17-4	39.23994406
Myxococcus fulvus	38.44913841
Desulfotomaculum acetoxidans	38.02951522
Exiguobacterium sp. AT1b	38.00303855
Providencia stuartii	37.99491934
Paenibacillus polymyxa	37.87647568
Primula malacoides virus 1	37.37674861
Stackebrandtia nassauensis	37.30173389
Kyrpidia tusciae	36.76373699
Ferrimonas balearica	36.40231604
Corynebacterium maris	35.91862023
Bifidobacterium breve	35.87853832
Lactobacillus acidophilus	35.84084255
Burkholderia glumae	35.27966601
Vibrio parahaemolyticus	35.13535972
Halobacillus halophilus	35.01348924
Mycoplasma bovis	34.57651941
Red clover cryptic virus 2	34.48640437
Wigglesworthia glossinidia	34.35560351
Mycobacterium rhodesiae	34.29675735
Pusillimonas sp. T7-7	34.06317717
Moorella thermoacetica	33.85157851
Dickeya phage RC-2014	33.5587078
Sideroxydans lithotrophicus	33.55868786
Shewanella baltica	33.24708287
Blattabacterium sp. (Blatta orientalis)	33.19650564
Bacillus cellulosilyticus	33.16598811
Psychroflexus torquis	33.12588626
Candidatus Profftella armatura	32.33533768
Oenococcus oeni	32.15245449
Streptomyces pratensis	31.85346386
secondary endosymbiont of Ctenarytaina eucalypti	31.7994498
Pyrolobus fumarii	31.41130504
candidate division SR1 bacterium RAAC1_SR1_1	31.39312487
Finegoldia magna	31.274494
Eggerthella sp. YY7918	31.09459095
Kytococcus sedentarius	30.99323686
Glaciecola psychrophila	30.68398803
Oceanobacillus iheyensis	30.66327441
Xanthomonas campestris	30.52342329
Helicobacter bizzozeronii	30.47429124
Alicyclobacillus acidocaldarius	30.34345039
Brevibacillus brevis	30.17440956
Sphaerochaeta globosa	29.92479812
Streptococcus oralis	29.7258937
Catenulispora acidiphila	28.99599332

Anoxybacillus flavithermus	28.90371935
Bifidobacterium asteroides	28.79193981
Fibrobacter succinogenes	28.74311472
Pantoea sp. At-9b	28.62220515
Verminephrobacter eiseniae	28.53745639
Halorhodospira halophila	28.26239905
Propionibacterium acidipropionici	28.12372602
Acinetobacter calcoaceticus	28.11978497
Proteus mirabilis	28.01216364
Ramlibacter tataouinensis	27.67271428
Laribacter hongkongensis	27.50825841
Photobacterium profundum	27.46214638
Burkholderia pseudomallei	27.28973856
Bacteroides helcogenes	27.18038504
Teredinibacter turnerae	26.56195724
Bordetella petrii	26.55129225
Streptococcus phage SM1	25.85719579
Staphylococcus warneri	25.55467092
Shigella flexneri	25.50886586
Dehalococcoides mccartyi	25.33272425
Clavibacter michiganensis	25.17431845
Bifidobacterium dentium	24.83015675
Halothermothrix orenii	24.78067774
Salivirus FHB	24.77618258
Enterobacter cloacae	24.71950538
Streptococcus phage YMC-2011	24.62233948
Bacteriovorax marinus	24.57271314
Pseudomonas stutzeri	24.24178978
Clostridiales genomosp. BVAB3	24.1929647
Leuconostoc carnosum	24.13570342
Solibacillus silvestris	23.758381
Oceanimonas sp. GK1	23.71012998
Deinococcus proteolyticus	23.64776951
Thermoanaerobacterium thermosaccharolyticum	23.36176015
Lactobacillus delbrueckii	23.13118006
Pandoravirus dulcis	23.1254569
Mycobacterium leprae	23.01933065
Candidatus Uzinura diaspidicola	22.64083015
Geobacter bemidjiensis	22.6245318
Corynebacterium resistens	22.46450124
Carnobacterium sp. WN1359	22.4467478
Thioalkalivibrio sp. K90mix	22.31858011
Klebsiella variicola	22.18298703
Acidaminococcus fermentans	22.17483786
Candidatus Riesia pediculicola	22.02747157
Dechloromonas aromatica	21.99215191
Streptococcus gallolyticus	21.93890152
Kosmotoga olearia	21.85331161
alpha proteobacterium HIMB5	21.81440779
Xylanimonas cellulosilytica	21.73873914
Legionella longbeachae	21.70949951
Truepera radiovictrix	21.59108581
Belliella baltica	21.48139534

Enterococcus mundtii	21.36985292
Rhodothermus marinus	21.36562484
Arthrobacter phenanthrenivorans	21.32736493
Leuconostoc mesenteroides	21.26258847
Frankia symbiont of Datisca glomerata	21.22159557
Acholeplasma brassicae	21.14708505
Anaerobaculum mobile	20.83999516
Bacillus pseudofirmus	20.4502955
Candidatus Endolissoclinum faulkneri	20.42708599
Candidatus Symbiobacter mobilis	20.2142893
Flavobacterium psychrophilum	20.21125926
Pseudomonas mendocina	20.08712241
Desulfarculus baarsii	19.97769903
Corynebacterium jeikeium	19.91080351
Thermobifida fusca	19.90839744
Shewanella loihica	19.88905913
Thermoanaerobacterium xylanolyticum	19.81947051
Staphylococcus carnosus	19.78032957
Lactobacillus sakei	19.76759544
Butyrivibrio fibrisolvens	19.76012014
Marinomonas sp. MWYL1	19.64136951
Enterococcus casseliflavus	19.46596161
[Clostridium] clariflavum	19.43133577
Isosphaera pallida	19.21488507
Clostridium beijerinckii	19.0994515
Paenibacillus sp. JDR-2	18.9316087
Corynebacterium aurimucosum	18.80019379
Malvastrum leaf curl Philippines betasatellite	18.6935034
Arthrobacter sp. FB24	18.61663672
Corynebacterium glutamicum	18.52321464
Tolumonas auensis	18.20010351
Listeria ivanovii	18.15252636
Sanguibacter keddieii	18.04037991
Bacillus megaterium	17.99576296
Pseudoalteromonas haloplanktis	17.99333694
Mycobacterium canettii	17.86408096
Magnetospirillum gryphiswaldense	17.84383165
Lawsonia intracellularis	17.64284807
Thermodesulfobium narugense	17.61872757
Desulfovibrio salexigens	17.60426124
Heliobacterium modesticaldum	17.27271391
Colwellia psychrerythraea	17.22299778
Sphaerobacter thermophilus	17.14190302
Nitratiruptor sp. SB155-2	17.02923242
Advenella kashmirensis	17.01865723
Streptosporangium roseum	17.0135381
Coxiella burnetii	17.00991405
Legionella pneumophila	16.99878474
Candidatus Kinetoplastibacterium galatii	16.78538403
Corynebacterium pseudotuberculosis	16.72540969
Haliangium ochraceum	16.61238221
Helicobacter pylori	16.4529257
Streptococcus phage Cp-1	16.30077722

Photorhabdus asymbiotica	16.2926081
Intrasporangium calvum	16.26458644
Hippea maritima	16.24344608
Burkholderia mallei	16.17837256
Rhodopseudomonas palustris	16.10241688
Fusobacterium periodonticum	16.02497614
Bifidobacterium bifidum	15.98609227
Clostridium pasteurianum	15.93212811
Moraxella catarrhalis	15.6413066
Candidatus Liberibacter solanacearum	15.16960119
Clostridium saccharobutylicum	15.06988189
Chthonomonas calidirosea	15.00957062
Nocardioides sp. JS614	14.86010522
Bacillus sp. 1NLA3E	14.85557017
Petrotoga mobilis	14.76092009
Klebsiella oxytoca	14.76002904
Geobacillus thermoglucosidasius	14.69250956
Cardinium endosymbiont of Encarsia pergandiella	14.68647943
Methylobacterium radiotolerans	14.6608838
Dictyoglomus thermophilum	14.50263533
Marinomonas posidonica	14.11596571
Psychromonas sp. CNPT3	13.87603842
Candidatus Tremblaya princeps	13.80431081
Pectobacterium carotovorum	13.59213809
Bacillus weihenstephanensis	13.51409332
Methylophaga frappieri	13.35946891
Muricauda ruestringensis	13.35192376
Cellulomonas flavigena	13.33834849
Clostridium novyi	13.31667395
Nocardiopsis dassonvillei	13.31426788
Trichodesmium erythraeum	13.23922319
Marinitoga piezophila	13.12528471
Polaribacter sp. MED152	13.10209515
Hirschia baltica	13.02039637
Anaerolinea thermophila	12.95439192
Erwinia amylovora	12.86457393
Methylobacterium extorquens	12.79978745
Opitutus terrae	12.72020772
Methanococcus aeolicus	12.69973121
Photorhabdus luminescens	12.62195353
Dill cryptic virus 1	12.39110635
Gloeobacter violaceus	12.37875907
Coprothermobacter proteolyticus	12.2681077
Blastococcus saxobsidens	12.24582914
Streptomyces venezuelae	12.22261964
Actinosynnema mirum	12.15149605
Marinobacter hydrocarbonoclasticus	12.11985035
Candidatus Hodgkinia cicadicola	12.08488757
Cyprinid herpesvirus 3	12.01919009
Agrobacterium vitis	11.98509842
Microcoleus sp. PCC 7113	11.91010365
Aliivibrio salmonicida	11.85402045
Desulfovibrio piezophilus	11.74913211

Symbiobacterium thermophilum	11.74399304
Candidatus Rickettsia amblyommii	11.73678483
Methylacidiphilum infernorum	11.70119809
Paenibacillus larvae	11.64573886
Streptomyces cattleya	11.60628092
Fervidobacterium nodosum	11.58126937
Thermanaerovibrio acidaminovorans	11.54027646
Bartonella grahamii	11.45163656
Gallionella capsiferriformans	11.38142398
Marinithermus hydrothermalis	11.29251699
Carp picornavirus 1	11.17710337
Pseudomonas putida	11.1626171
Hydrogenobaculum sp. HO	11.16019108
Salinispora tropica	11.1412296
Idiomarina loihiensis	11.13428847
Thermodesulfobacterium geofontis	11.00861666
Plautia stali symbiont	10.98389213
Streptococcus phage PH10	10.90640148
Geobacter uraniireducens	10.81631641
Rhodococcus erythropolis	10.754847
Klebsiella pneumoniae	10.72740943
Streptomyces avermitilis	10.7232013
[Cellvibrio] gilvus	10.69667472
Acidimicrobium ferrooxidans	10.68884255
Cellulophaga algicola	10.57672606
Staphylococcus saprophyticus	10.57160693
Thermoanaerobacter wiegelii	10.55985366
Burkholderia sp. KJ006	10.53512913
Wolbachia endosymbiont of Onchocerca ochengi	10.20689888
Bifidobacterium longum	10.16895596
Sulfuricella denitrificans	10.09478239
Fretibacterium fastidiosum	10.07608799
Prevotela denticola	10.05109638
Vibrio vulnificus	10.04506625
Spirochaeta smaragdinae	9.960674371
Candidatus Blochmannia floridanus	9.922711513
Gordonia polyisoprenivorans	9.822942305
Lactobacillus johnsonii	9.7587399
Mesotoga prima	9.729520217
Singulisphaera acidiphila	9.713538868
Slackia heliotrinireducens	9.665604841
Enterococcus faecalis	9.627037959
Gardnerella vaginalis	9.585154001
Candidatus Tremblaya phenacola	9.581816983
Emticicia oligotrophica	9.530298796
Zymomonas mobilis	9.510356459
Rickettsia africae	9.498049068
Spirosoma linguale	9.486899816
Phycisphaera mikurensis	9.446797967
Ornithobacterium rhinotracheale	9.350058804
Saccharomonospora viridis	9.310867953
Psychromonas ingrahamii	9.268072998
Delftia acidovorans	9.263864869

Flexibacter litoralis	9.215306874
Zunongwangia profunda	9.173759856
Oscillatoria nigro-viridis	9.160471612
Human endogenous retrovirus K	9.126132803
Coriobacterium glomerans	9.112270501
Bacillus pumilus	9.106527405
Geobacter lovleyi	9.099319197
Thermobacillus composti	9.059504377
Mycoplasma gallisepticum	9.037829842
Tepidanaerobacter acetatoxydans	8.966993287
Pirellula staleyi	8.920881257
Desulfovibrio vulgaris	8.91576213
Syntrophus aciditrophicus	8.863332946
Ureaplasma parvum	8.842836494
Nitrosomonas eutropha	8.693618237
Enterobacter lignolyticus	8.656853406
Actinoplanes friuliensis	8.634554903
Ralstonia pickettii	8.631237828
Corynebacterium ulcerans	8.631237828
Azospirillum brasilense	8.588709958
Lactobacillus crispatus	8.55651014
Shewanella sp. W3-18-1	8.524864436
Leuconostoc citreum	8.51669532
Clostridium sp. BNL1100	8.496198868
Arcobacter butzleri	8.435333482
Shewanella piezotolerans	8.373844127
Dehalogenimonas lykanthroporepellens	8.283135089
Campylobacter coli	8.281333037
Spiroplasma chrysopicola	8.279530985
Desulfurobacterium thermolithotrophum	8.21832866
Weeksella virosa	8.21804163
Caldicellulosiruptor obsidiansis	8.165899475
Mycobacterium chubuense	8.081507592
Pseudomonas resinovorans	8.0739425
Lactobacillus brevis	8.01218606
Corynebacterium halotolerans	7.878058098
Thermovirga lienii	7.790666135
Chromohalobacter salexigens	7.781318935
Herbaspirillum seropedicae	7.764426588
Arthrobacter chlorophenolicus	7.689094875
Desulfotomaculum reducens	7.684866802
Rhodococcus pyridinivorans	7.602880996
Delftia sp. Cs1-4	7.594157764
Cyprinid herpesvirus 1	7.535985484
Caldicellulosiruptor bescii	7.484754327
Microlunatus phosphovorus	7.469377002
Aequorivita sublithincola	7.447078499
Candidatus Solibacter usitatus	7.416947818
Deinococcus maricopensis	7.411204722
Gordonia sp. KTR9	7.346398293
Cellvibrio japonicus	7.311148485
Burkholderia cenocepacia	7.27106658
Francisella tularensis	7.263234403

Acidithiobacillus ferrivorans	7.204438154
Acholeplasma laidlawii	7.201745048
Halanaerobium praevalens	7.18213965
Streptococcus phage Sfi21	7.181515681
Burkholderia rhizoxinica	7.116155137
Serratia marcescens	7.072736213
Syntrophothermus lipocalidus	7.061586961
Roseobacter denitrificans	7.037486405
Pantoea vagans	7.011870827
Candidatus Cloacimonas acidaminovorans	7.000097606
Azospirillum lipoferum	6.95129247
Parvularcula bermudensis	6.855444361
Formica exsecta virus 1	6.826511708
Pandoravirus salinus	6.783716752
Aquifex aeolicus	6.747842976
Burkholderia thailandensis	6.674313315
Lactobacillus gasseri	6.642667612
Shewanella pealeana	6.641756614
[Ruminococcus] obeum	6.637528541
Gloeobacter kilaueensis	6.61011091
Ruegeria sp. TM1040	6.608308858
Burkholderia lata	6.590525456
Thermincola potens	6.439218118
Burkholderia phytofirmans	6.405146394
Croceibacter atlanticus	6.371698638
Xylella fastidiosa	6.3090312
Calditerrivibrio nitroreducens	6.299684
Rickettsia canadensis	6.288534748
Ruminococcus champanellensis	6.277672526
Acidaminococcus intestini	6.238194645
Listeria welshimeri	6.158634859
Bacillus halodurans	6.037458201
Cupriavidus metallidurans	5.989544119
Mycoplasma putrefaciens	5.922024639
Gemmatimonas aurantiaca	5.861159253
butyrate-producing bacterium SS3/4	5.838236781
Chlamydia pneumoniae	5.724605271
Paenibacillus terrae	5.70653484
Actinoplanes sp. N902-109	5.679117209
Natranaerobius thermophilus	5.59290333
Nitrosomonas sp. Is79A3	5.551019372
Burkholderia ambifaria	5.53987012
Geobacter metallireducens	5.539583091
Rahnella aquatilis	5.532037943
secondary endosymbiont of Heteropsylla cubana	5.513343544
Bifidobacterium animalis	5.471459586
Paenibacillus sp. Y412MC10	5.429555685
Glaciecola nitratireducens	5.418406433
Mycoplasma mobile	5.390988803
Brucella melitensis	5.370492351
Thalassolituus oleivorans	5.345767826
Asticcacaulis excentricus	5.335529573
Desulfurivibrio alkaliphilus	5.29424964

Desulfohalobium retbaense	5.29424964
Maricaulis maris	5.258662893
Bradyrhizobium sp. S23321	5.247513641
Methylobacterium populi	5.246622587
Corynebacterium callunae	5.225215137
Desulfotomaculum ruminis	5.215867938
Acinetobacter oleivorans	5.210748811
Methylococcus capsulatus	5.199599559
Enterobacter asburiae	5.18061813
Streptococcus phage IC1	5.18061813
Rubrivivax gelatinosus	5.163725782
Methanosarcina mazei	5.090196122
Thermotoga thermarum	5.084165997
Adlercreutzia equolifaciens	5.028419737
Bradyrhizobium diazoefficiens	4.995259011
Chlamydia psittaci	4.993456959
Clostridium phage c-st	4.985624782
Erwinia tasmaniensis	4.978079634
Rickettsia bellii	4.96512833
Desulfosporosinus orientis	4.943116856
Kangiella koreensis	4.924135427
Halothece sp. PCC 7418	4.918392331
Cytophaga hutchinsonii	4.84214962
Sulfurospirillum deleyianum	4.832802421
Pseudomonas fluorescens	4.825881242
Phenylobacterium zucineum	4.743895435
Gordonibacter pamelaeae	4.734548235
Nitrosococcus oceani	4.688149176
Streptomyces albus	4.68240608
Cellulomonas fimi	4.666137701
Thermodesulfatator indicus	4.623342746
Nostoc sp. PCC 7120	4.619114673
[Clostridium] cellulolyticum	4.576319718
Persephonella marina	4.560051339
Lactobacillus plantarum	4.539554887
Streptomyces coelicolor	4.525088561
Chroococcidiopsis thermalis	4.507909184
uncultured Termite group 1 bacterium	4.487412732
Stanieria cyanosphaera	4.483184659
Candidatus Liberibacter asiaticus	4.441300702
Bacillus infantis	4.403624873
Sorangium cellulosum	4.392475622
Synechococcus sp. PCC 7502	4.360205949
Anabaena sp. 90	4.346363591
Pseudoalteromonas atlantica	4.342135519
Elusimicrobium minutum	4.269496912
Bradyrhizobium japonicum	4.236960154
Solenopsis invicta virus 3	4.225810902
Saprospira grandis	4.206205505
Candidatus Moranella endobia	4.199284326
Bradyrhizobium sp. BTAi1	4.184817999
Ruminococcus albus	4.162519495
Methanospirillum hungatei	4.162519495

Nocardia cyriacigeorgica	4.142023044
Desulfotomaculum carboxydivorans	4.137794971
Leuconostoc gasicomitatum	4.137794971
Starkeya novella	4.135992919
Thiomicrospira crunogena	4.135992919
Acetohalobium arabaticum	4.104347215
Candidatus Arthromitus sp. SFB-mouse	4.089880888
Ruminococcus bromii	4.04285786
Nakamurella multipartita	4.04285786
Vibrio anguillarum	4.022361409
Lactobacillus rhamnosus	4.00609303
Syntrophobotulus glycolicus	3.953950875
Pediococcus pentosaceus	3.922305171
Blattabacterium sp. (Blaberus giganteus)	3.922305171
Bacillus clausii	3.890659468
Nitrosomonas europaea	3.867736995
Methylotenera versatilis	3.857211712
Alkaliphilus oremlandii	3.847864512
Turneriella parva	3.83671526
Calyptogena okutanii thioautotrophic gill symbiont	3.833398186
Candidatus Sulfuricurvum sp. RIFRC-1	3.819822913
Mycoplasma fermentans	3.786375157
Herminiimonas arsenicoxydans	3.777027958
Dokdonia sp. 4H-3-7-5	3.745382254
Shewanella woodyi	3.740263127
Candidatus Phytoplasma mali	3.729113875
Rhodococcus hoagii	3.729113875
Francisella sp. TX077308	3.664020416
Aureococcus anophagefferens virus	3.651356142
Anabaena variabilis	3.647128069
Deferribacter desulfuricans	3.645326017
Alicycliphilus denitrificans	3.645326017
Lactobacillus buchneri	3.643523965
Xanthobacter autotrophicus	3.619710438
Candidatus Amoebophilus asiaticus	3.611878261
Spirochaeta africana	3.611254292
Acidovorax sp. JS42	3.611254292
White clover cryptic virus 2	3.608561186
Nitrosococcus halophilus	3.590757841
Rhodococcus opacus	3.588064735
Nitrosomonas sp. AL212	3.583836662
Saccharophagus degradans	3.58203461
Bacillus anthracis	3.58203461
Dactylococcopsis salina	3.509396003
Candidatus Portiera aleyrodidarum	3.488899551
Vibrio sp. Ex25	3.463283973
Campylobacter lari	3.457253848
Bartonella australis	3.452134721
Candidatus Nasuia deltocephalinicola	3.440985469
Yersinia pestis	3.388843314
Pseudomonas syringae group genomosp. 3	3.375268041
Burkholderia cepacia	3.363227735
Rhodospirillum centenum	3.354771589

candidate division WWE3 bacterium RAAC2_WWE3_1	3.336701159
Halomonas elongata	3.334899106
Thioalkalivibrio nitratireducens	3.327353959
Methylobacillus flagellatus	3.325551907
Clostridium saccharoperbutylacetonicum	3.284559003
Kitasatospora setae	3.284559003
Raoultella ornithinolytica	3.2529133
Mycoplasma pulmonis	3.247794173
Brachyspira murdochii	3.241764048
Cupriavidus necator	3.232416848
Desulfovibrio desulfuricans	3.200771145
Streptococcus phage EJ-1	3.200771145
Actinoplanes sp. SE50/110	3.195652018
Herpetosiphon aurantiacus	3.180274693
Desulfotomaculum gibsoniae	3.150431042
Robiginitalea biformata	3.134162663
Melioribacter roseus	3.118785338
Spirochaeta sp. L21-RPul-D2	3.110062107
Acinetobacter baumannii	3.059722004
Rivularia sp. PCC 7116	3.059722004
Amycolatopsis orientalis	3.054602877
Helicobacter hepaticus	3.016927049
Candidatus Ruthia magnifica	3.007579849
Exiguobacterium sp. MH3	3.005777797
Borrelia recurrentis	2.987083397
Burkholderia sp. YI23	2.98196427
Calothrix sp. PCC 7507	2.975934145
Falconid herpesvirus 1	2.970815018
Methylibium petroleiphilum	2.929822115
Pseudomonas entomophila	2.929822115
Shewanella sp. ANA-3	2.928020063
Desulfococcus oleovorans	2.903295539
Pediococcus claussenii	2.875877908
Mycobacterium gilvum	2.866530708
Deinococcus radiodurans	2.844232204
Synechococcus sp. JA-2-3B'a(2-13)	2.823735753
Streptococcus phage Abc2	2.821309732
Rhodomicrobium vannielii	2.801437249
Polaromonas sp. JS666	2.793892101
Vibrio furnissii	2.769791545
Phaseolus vulgaris endornavirus	2.769791545
Orientia tsutsugamushi	2.749295094
Echinicola vietnamensis	2.728798642
Pectobacterium atrosepticum	2.710104243
Acidovorax sp. KKS102	2.673339412
Methylobacterium nodulans	2.626316384
Aeromonas salmonicida	2.616969184
Saccharothrix espanaensis	2.56725305
Desulfotomaculum kuznetsovii	2.535607346
Erwinia sp. Ejp617	2.535607346
Listeria innocua	2.515110895
Desulfosporosinus meridiei	2.503961643
Shewanella frigidimarina	2.492812391

Enterococcus sp. 7L76	2.492812391
Arthrobacter sp. Rue61a	2.472315939
Nostoc punctiforme	2.46296874
Vibrio tasmaniensis	2.46296874
Nonlabens dokdonensis	2.451819488
Methanosarcina barkeri	2.437353161
Alistipes finegoldii	2.421975836
Candidatus Accumulibacter phosphatis	2.421975836
Paramecium bursaria Chlorella virus 1	2.405707457
Geobacter sp. M18	2.385211006
Xanthomonas albilineans	2.23813174
Leptospira borgpetersenii	2.206486037
Pseudomonas syringae	2.206486037
Geobacillus sp. C56-T3	2.197138837
Escherichia fergusonii	2.197138837
Marinobacter sp. BSs20148	2.176642385
Micavibrio aeruginosavorus	2.176642385
Helicobacter felis	2.174840333
Eggerthella lenta	2.160374006
Leisingera methylohalidivorans	2.160374006
Pantoea ananatis	2.13384743
Elephantid herpesvirus 1	2.128728303
Hyphomonas neptunium	2.12450023
Acidithiobacillus caldus	2.113350978
Polaromonas naphthalenivorans	2.076586148
Polynucleobacter necessarius	2.065436896
Methanosarcina acetivorans	2.065436896
Dickeya zeae	2.056089696
Shewanella oneidensis	2.044940444
gamma proteobacterium HdN1	2.044940444
Carboxydothermus hydrogenoformans	2.013294741
Oligotropha carboxidovorans	1.951805386
Oscillatoria acuminata	1.940656134
Enterobacter sp. 638	1.929506882
Ignavibacterium album	1.90901043
Desulfobacca acetoxidans	1.90901043
Leptospira biflexa	1.90901043
Sulfuricurvum kujiense	1.888513979
Arcobacter nitrofigilis	1.877364727
Pseudoalteromonas sp. SM9913	1.877364727
Granulicella tundricola	1.868017527
Fluviicola taffensis	1.856868275
Alteromonas sp. SN2	1.856868275
Streptomyces hygroscopicus	1.851749148
Maruca vitrata nucleopolyhedrovirus	1.851749148
Spiroplasma taiwanense	1.851749148
Human herpesvirus 6B	1.851749148
Bordetella avium	1.836371824
Citrobacter koseri	1.820103445
Cyanothece sp. PCC 7424	1.79537892
Desulfocapsa sulfexigens	1.79537892
Arthrospira platensis	1.79537892
Halovivax ruber	1.788457741

Burkholderia sp. CCGE1002	1.76796129
Bradyrhizobium oligotrophicum	1.76796129
Methylomonas methanica	1.756812038
Azotobacter vinelandii	1.756812038
Caulobacter sp. K31	1.756812038
Aeromonas veronii	1.747464838
Edwardsiella ictaluri	1.747464838
Gordonia bronchialis	1.747464838
Cupriavidus pinatubonensis	1.736315586
Thermosynechococcus sp. NK55a	1.715819135
Paenibacillus mucilaginosus	1.684173431
Mycobacterium bovis	1.684173431
Enterobacter aerogenes	1.673024179
Mycoplasma arthritidis	1.661874927
Rhodobacter capsulatus	1.661874927
Brachyspira pilosicoli	1.661874927
Mycoplasma parvum	1.652527728
Brevundimonas subvibrioides	1.652527728
Chloroherpeton thalassium	1.641378476
Bordetella pertussis	1.632031276
Rhodopirellula baltica	1.632031276
Methylotenera mobilis	1.620882024
Thermosynechococcus elongatus	1.611534824
Conexibacter woesei	1.611534824
Hydrogenobaculum sp. Y04AAS1	1.611534824
Coraliomargarita akajimensis	1.611534824
Treponema caldaria	1.600385572
Clostridium ljungdahlii	1.600385572
Glaciecola sp. 4H-3-7+YE-5	1.559392669
Alcanivorax borkumensis	1.559392669
Rhizobium sp. IRBG74	1.54312429
Methanothermococcus okinawensis	1.54312429
Streptococcus phage Sfi19	1.538896217
Streptomyces scabiei	1.511478587
Salinispora arenicola	1.511478587
Burkholderia xenovorans	1.511478587
Rhizoctonia solani dsRNA virus 2	1.490982135
Geobacillus sp. WCH70	1.479832883
Rose rosette virus	1.479832883
Bartonella henselae	1.479832883
Pelagibacterium halotolerans	1.459336432
Choristoneura rosaceana entomopoxvirus 'L'	1.44818718
Shewanella amazonensis	1.43883998
Erythrobacter litoralis	1.43883998
Bradyrhizobium sp. ORS 278	1.43883998
Nitrosospira multiformis	1.427690728
Mycoplasma synoviae	1.427690728
Azorhizobium caulinodans	1.427690728
Geobacillus thermodenitrificans	1.416541476
Sinorhizobium fredii	1.416541476
Rhodoferax ferrireducens	1.396045025
Staphylococcus pseudintermedius	1.396045025
Listeria seeligeri	1.384895773

Bacillus atrophaeus	1.384895773
Cronobacter sakazakii	1.375548573
Stigmatella aurantiaca	1.375548573
Arcobacter sp. L	1.364399321
Bacteroides xylanisolvens	1.343902869
Desulfobacula toluolica	1.343902869
Blattabacterium sp. (Panesthia angustipennis spadica)	1.343902869
Corallococcus coralloides	1.33455567
Desulfitobacterium hafniense	1.33455567
Xenorhabdus bovienii	1.323406418
Candidatus Babela massiliensis	1.323406418
Vibrio nigripulchritudo	1.323406418
Neorickettsia sennetsu	1.302909966
Streptomyces fulvissimus	1.302909966
Janthinobacterium sp. Marseille	1.282413515
Akkermansia muciniphila	1.282413515
Burkholderia sp. CCGE1003	1.234499432
Haliscomenobacter hydrossis	1.202853729
Emiliania huxleyi virus 86	1.202853729
Frankia sp. CcI3	1.202853729
Erwinia billingiae	1.202853729
Azospira oryzae	1.182357277
Burkholderia vietnamiensis	1.182357277
Corynebacterium terpenotabidum	1.182357277
Desulfatibacillum alkenivorans	1.171208025
Serratia proteamaculans	1.171208025
Cupriavidus taiwanensis	1.150711574
Streptomyces collinus	1.150711574
Candidatus Kinetoplastibacterium desouzaii	1.150711574
Geodermatophilus obscurus	1.139562322
Roseburia intestinalis	1.130215122
Xenorhabdus nematophila	1.130215122
Pseudoxanthomonas suwonensis	1.130215122
Waddlia chondrophila	1.130215122
Candidatus Midichloria mitochondrii	1.130215122
Fibrella aestuarina	1.11906587
Thermacetogenium phaeum	1.107916618
Beijerinckia indica	1.107916618
Streptococcus phage DCC1738	1.107916618
Bartonella clarridgeiae	1.107916618
Pectobacterium sp. SCC3193	1.107916618
Streptococcus intermedius	1.098569418
Planctomyces brasiliensis	1.098569418
Citrobacter rodentium	1.098569418
Synechococcus sp. RCC307	1.087420167
Mycoplasma penetrans	1.087420167
Psychrobacter sp. PRwf-1	1.087420167
Maribacter sp. HTCC2170	1.087420167
Runella slithyformis	1.087420167
Bacillus licheniformis	1.087420167
Caldisericum exile	1.087420167
Methylovorus sp. MP688	1.087420167
Desulfovibrio gigas	1.087420167

Candidatus Pelagibacter sp. IMCC9063	1.078072967
Enterobacteriaceae bacterium strain FGI 57	1.078072967
Methylocystis sp. SC2	1.066923715
Pseudovibrio sp. FO-BEG1	1.066923715
Candidatus Arthromitus sp. SFB-rat-Yit	1.066923715
Lacinutrix sp. 5H-3-7-4	1.066923715
Spiroplasma apis	1.066923715
Thermobaculum terrenum	1.046427263
Frankia sp. EAN1pec	1.046427263
Synechococcus sp. JA-3-3Ab	1.046427263
Thermaerobacter marianensis	1.046427263
Streptococcus phage phiBHN167	1.046427263
Sphingomonas sp. MM-1	1.025930812
Morganella morganii	1.025930812
Shewanella violacea	1.025930812
Desulfitobacterium dehalogenans	1.025930812
Synechococcus elongatus	1.025930812
Vicia cryptic virus	0.925874574
Ehrlichia ruminantium	0.925874574
Rhodococcus jostii	0.925874574
Cryptophlebia leucotreta granulovirus	0.925874574
Bdellovibrio exovorus	0.925874574
Hyposoter fugitivus ichnovirus	0.894228871
Pelotomaculum thermopropionicum	0.894228871
Pandoraea sp. RB-44	0.894228871
beta proteobacterium CB	0.894228871
Pseudomonas sp. VLB120	0.894228871
Methanobrevibacter ruminantium	0.894228871
Laodelphax striatella honeydew virus 1	0.873732419
Rhodobacter sphaeroides	0.873732419
Borrelia crocidurae	0.873732419
Sphingopyxis alaskensis	0.873732419
Bdellovibrio bacteriovorus	0.862583167
Cyanobium gracile	0.862583167
Yersinia pseudotuberculosis	0.862583167
Desulfovibrio magneticus	0.862583167
Trichormus azollae	0.862583167
Candidatus Pelagibacter ubique	0.862583167
Sulfurovum sp. NBC37-1	0.842086716
Streptomyces davawensis	0.842086716
Human herpesvirus 7	0.842086716
Cyprinid herpesvirus 2	0.842086716
Nitrobacter winogradskyi	0.842086716
Cynomolgus macaque cytomegalovirus strain Ottawa	0.842086716
Verrucosispora maris	0.842086716
Hyphomicrobium denitrificans	0.842086716
Methanobrevibacter sp. AbM4	0.842086716
Thermotoga lettingae	0.830937464
Streptococcus phage K13	0.830937464
Geobacillus sp. JF8	0.830937464
Candidatus Desulforudis audaxviator	0.821590264
Enterobacter sp. R4-368	0.821590264
Paracoccus denitrificans	0.821590264

Halyomorpha halys symbiont	0.810441012
Anaeromyxobacter dehalogenans	0.810441012
Thioflavicoccus mobilis	0.810441012
Staphylothermus hellenicus	0.810441012
Blattabacterium punctulatus	0.78994456
Rhizobium leguminosarum	0.78994456
Nautilia profundicola	0.78994456
Thermodesulfovibrio yellowstonii	0.78994456
Octadecabacter antarcticus	0.78994456
Staphylococcus pasteuri	0.78994456
Streptomyces violaceusniger	0.769448109
Geobacter sulfurreducens	0.769448109
Sulfurimonas autotrophica	0.769448109
Myxococcus xanthus	0.769448109
Tistrella mobilis	0.769448109
Acinetobacter sp. ADP1	0.617249716
Papaya leaf curl alphasatellite	0.617249716
Cercopithecine herpesvirus 2	0.617249716
Sphingobium sp. SYK-6	0.617249716
Pseudomonas fulva	0.617249716
Pseudomonas poae	0.617249716
Thiomonas intermedia	0.617249716
Chlorobium phaeovibrioides	0.617249716
Sphaerochaeta pleomorpha	0.617249716
Bordetella bronchiseptica	0.617249716
Rosellinia necatrix partitivirus 2	0.617249716
Chlamydia pecorum	0.617249716
Invertebrate iridescent virus 31	0.617249716
Caldisphaera lagunensis	0.617249716
Cucumber green mottle mosaic virus	0.617249716
Haemophilus phage HP1	0.617249716
Impatiens necrotic spot virus	0.617249716
Thermocrinis albus	0.617249716
Shewanella halifaxensis	0.585604013
Dyadobacter fermentans	0.585604013
Azoarcus sp. KH32C	0.585604013
Flavobacterium johnsoniae	0.585604013
Phaeocystis globosa virus	0.585604013
Spiribacter sp. UAH-SP71	0.585604013
Fervidobacterium pennivorans	0.585604013
Psychrobacter arcticus	0.585604013
Mycoplasma conjunctivae	0.585604013
Alcanivorax dieselolei	0.585604013
Leptospira interrogans	0.585604013
Streptococcus phage PH15	0.585604013
Shewanella denitrificans	0.565107561
Methylobacterium sp. 4-46	0.565107561
Methanococcus vannielii	0.565107561
Methanocella paludicola	0.565107561
Desulfovibrio africanus	0.553958309
Spiribacter salinus	0.553958309
Desulfovibrio alaskensis	0.553958309
Simiduia agarivorans	0.553958309

Agrobacterium fabrum	0.553958309
Pseudomonas protegens	0.553958309
Chloroflexus aggregans	0.553958309
Candidatus Kinetoplastibacterium crithidii	0.553958309
Thermoplasma volcanium	0.553958309
Mycoplasma agalactiae	0.553958309
Ectocarpus siliculosus virus 1	0.553958309
Prosthecochloris aestuarii	0.553958309
Candidatus Atelocyanobacterium thalassa	0.553958309
Geobacillus thermoleovorans	0.553958309
Lactobacillus paracasei	0.553958309
Vibrio alginolyticus	0.553958309
Helicobacter cetorum	0.553958309
Human herpesvirus 2	0.553958309
Mycoplasma pneumoniae	0.553958309
Oryctes rhinoceros nudivirus	0.553958309
Serratia plymuthica	0.553958309
Sinorhizobium meliloti	0.553958309
Burkholderia phymatum	0.533461857
Nitratifractor salsuginis	0.533461857
Deinococcus geothermalis	0.533461857
Mycoplasma mycoides	0.533461857
Wolinella succinogenes	0.533461857
Aeromonas hydrophila	0.533461857
Anabaena cylindrica	0.533461857
Sphaerochaeta coccoides	0.533461857
Mycobacterium ulcerans	0.512965406
Modestobacter marinus	0.512965406
Bartonella quintana	0.512965406
Thermomicrobium roseum	0.512965406
Aeromonas phage phiAS5	0.512965406
Paracoccus aminophilus	0.512965406
Salmonella bongori	0.512965406
Thermotoga maritima	0.512965406
Synechococcus sp. PCC 6312	0.512965406
Acidiphilium multivorum	0.512965406
Gluconacetobacter diazotrophicus	0.512965406
Myxococcus stipitatus	0.512965406
Pseudanabaena sp. PCC 7367	0.308624858
Pepino mosaic virus	0.308624858
Burkholderia sp. RPE64	0.308624858
Methanococcus voltae	0.308624858
Lactobacillus helveticus	0.308624858
Xanthomonas oryzae	0.308624858
Sphingomonas wittichii	0.308624858
Granulicella mallensis	0.308624858
Yersinia phage phiA1122	0.308624858
Actinoplanes missouriensis	0.308624858
Pseudoxanthomonas spadix	0.308624858
Ictalurid herpesvirus 1	0.308624858
Eel picornavirus 1	0.308624858
Pelobacter carbinolicus	0.308624858
Acidovorax citrulli	0.308624858

Aquamavirus A	0.308624858
Erinnyis ello granulovirus	0.308624858
Marinomonas mediterranea	0.308624858
Streptomyces sp. SirexAA-E	0.308624858
Caulobacter vibrioides	0.308624858
Mycobacterium avium	0.308624858
Thermoanaerobacter italicus	0.308624858
Rickettsia akari	0.308624858
Glypta fumiferanae ichnovirus	0.308624858
Grapevine Syrah virus 1	0.308624858
Marinobacter adhaerens	0.308624858
Saimiriine herpesvirus 1	0.308624858
Rhizobium etli	0.308624858
Mesorhizobium opportunistum	0.308624858
Agrobacterium sp. H13-3	0.308624858
Candidatus Blochmannia vafer	0.308624858
Caulobacter segnis	0.308624858
Cotesia congregata bracovirus	0.308624858
Francisella noatunensis	0.308624858
Human herpesvirus 5	0.308624858
Human mastadenovirus C	0.308624858
Leuconostoc sp. C2	0.308624858
Natronococcus occultus	0.308624858
Rice grassy stunt virus	0.308624858
White spot syndrome virus	0.308624858
Invertebrate iridescent virus 6	0.308624858
Pelobacter propionicus	0.308624858
Acanthamoeba polyphaga mimivirus	0.308624858
Alcelaphine herpesvirus 2	0.308624858
Bacillus phage phiNIT1	0.308624858
Carnivore amdoparvovirus 1	0.308624858
Deinococcus gobiensis	0.308624858
Enterobacteria phage lambda	0.308624858
Halalkalicoccus jeotgali	0.308624858
Mesorhizobium loti	0.308624858
Micromonospora aurantiaca	0.308624858
Tanapox virus	0.308624858
Ureaplasma urealyticum	0.308624858
Candidatus Protochlamydia amoebophila	0.276979155
Mesoplasma florum	0.276979155
Pleurocapsa minor	0.276979155
Desulfosporosinus acidiphilus	0.276979155
Pseudomonas sp. UW4	0.276979155
Acidovorax avenae	0.276979155
Melon yellow spot virus	0.276979155
Novosphingobium sp. PP1Y	0.276979155
Thermus scotoductus	0.276979155
Helicobacter mustelae	0.276979155
Sphingobium japonicum	0.276979155
Blattabacterium sp. (Blattella germanica)	0.276979155
Pseudomonas monteilii	0.276979155
Methylovorus glucosotrophus	0.276979155
Frankia alni	0.276979155

Neorickettsia risticii	0.276979155
Bartonella bacilliformis	0.276979155
Macacine herpesvirus 1	0.276979155
Serratia liquefaciens	0.276979155
Francisella philomiragia	0.276979155
Rickettsia rhipicephali	0.276979155
Synechococcus sp. WH 7803	0.276979155
Burkholderia phage Bcep22	0.276979155
Crinalium epipsammum	0.276979155
Cronobacter turicensis	0.276979155
Cyanothece sp. ATCC 51142	0.276979155
Cylindrospermum stagnale	0.276979155
Hibiscus latent Singapore virus	0.276979155
Mycoplasma wenyonii	0.276979155
Xanthomonas fuscans	0.276979155
Thermus thermophilus	0.256482703
Salinarchaeum sp. Harcht-Bsk1	0.256482703
Pseudomonas brassicacearum	0.256482703
Terriglobus roseus	0.256482703
Arthrobacter aurescens	0.256482703
Hyphomicrobium sp. MC1	0.256482703
Nocardia brasiliensis	0.256482703
Mycobacterium vanbaalenii	0.256482703
Burkholderia phenoliruptrix	0.256482703
Blattabacterium sp. (Periplaneta americana)	0.256482703
Sulfurospirillum barnesii	0.256482703
Deinococcus peraridilitoris	0.256482703
Pseudomonas sp. TKP	0.256482703
Brachyspira hyodysenteriae	0.256482703
Stenotrophomonas phage phiSMA7	0.256482703
Pelodictyon luteolum	0.256482703
Bacillus sp. JS	0.256482703
Agrobacterium tumefaciens	0.256482703
Acetobacter pasteurianus	0.256482703
Chlorobaculum parvum	0.256482703
Erwinia phage Ea35-70	0.256482703
Micromonospora sp. L5	0.256482703
Pectobacterium wasabiae	0.256482703
Psychrobacter sp. G	0.256482703
Streptococcus phage 7201	0.256482703
Terriglobus saanensis	0.256482703
Thiomonas arsenitoxydans	0.256482703

